# KLF2 regulates neutrophil activation and thrombosis in cardiac hypertrophy and heart failure progression

**DOI:** 10.1172/JCI147191

**Published:** 2022-02-01

**Authors:** Xinmiao Tang, Peiwei Wang, Rongli Zhang, Ippei Watanabe, Eugene Chang, Vinesh Vinayachandran, Lalitha Nayak, Stephanie Lapping, Sarah Liao, Annmarie Madera, David R. Sweet, Jiemeng Luo, Jinsong Fei, Hyun-Woo Jeong, Ralf H. Adams, Teng Zhang, Xudong Liao, Mukesh K. Jain

**Affiliations:** 1 Yueyang Hospital, Shanghai University of Traditional Chinese Medicine, Shanghai, China.; 2 Case Cardiovascular Research Institute, Case Western Reserve University School of Medicine, Harrington Heart and Vascular Institute, University Hospitals Cleveland Medical Center, Cleveland, Ohio, USA.; 3 Clinical Research Institute of Integrative Medicine, Shanghai Academy of Traditional Chinese Medicine, Shanghai, China.; 4 Minhang Hospital of Integrated Traditional Chinese and Western Medicine, Shanghai, China.; 5 Max Planck Institute for Molecular Biomedicine, Department of Tissue Morphogenesis, Münster, Germany.

**Keywords:** Cardiology, Inflammation, Cardiovascular disease, Neutrophils, Transcription

## Abstract

It is widely recognized that inflammation plays a critical role in cardiac hypertrophy and heart failure. However, clinical trials targeting cytokines have shown equivocal effects, indicating the need for a deeper understanding of the precise role of inflammation and inflammatory cells in heart failure. Leukocytes from human subjects and a rodent model of heart failure were characterized by a marked reduction in expression of *Klf2* mRNA. Using a mouse model of angiotensin II–induced nonischemic cardiac dysfunction, we showed that neutrophils played an essential role in the pathogenesis and progression of heart failure. Mechanistically, chronic angiotensin II infusion activated a neutrophil KLF2/NETosis pathway that triggered sporadic thrombosis in small myocardial vessels, leading to myocardial hypoxia, cell death, and hypertrophy. Conversely, targeting neutrophils, neutrophil extracellular traps (NETs), or thrombosis ameliorated these pathological changes and preserved cardiac dysfunction. KLF2 regulated neutrophil activation in response to angiotensin II at the molecular level, partly through crosstalk with HIF1 signaling. Taken together, our data implicate neutrophil-mediated immunothrombotic dysregulation as a critical pathogenic mechanism leading to cardiac hypertrophy and heart failure. This neutrophil KLF2-NETosis-thrombosis mechanism underlying chronic heart failure can be exploited for therapeutic gain by therapies targeting neutrophils, NETosis, or thrombosis.

## Graphical abstract

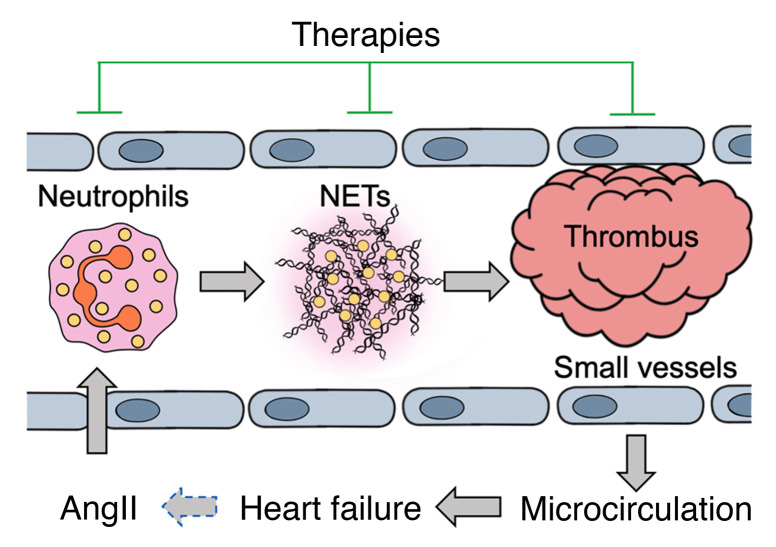

## Introduction

Although the development and progression of cardiac hypertrophy and failure have traditionally been viewed as hemodynamic and neurohormonal disorders, there is increasing awareness that inflammation also plays a critical role (1, 2). More than 3 centuries ago, physicians observed myocardial inflammation in the diseased heart (1). In the 1950s, C-reactive protein (CRP) was first identified as an inflammatory biomarker for acute myocardial infarction (AMI), and decades later, it was also shown to be associated with cardiac hypertrophy and failure (3–7). More recently, studies have found that proinflammatory cytokines, such as TNF-α, and members of the interleukin 1 (IL-1) and IL-6 families are elevated in patients with heart failure (HF). Further, experimental overexpression of these cytokines to clinically relevant levels drives cardiac hypertrophy and failure in animal models (8, 9). Hence, it was hypothesized that elevated proinflammatory cytokines are significant contributors to cardiac hypertrophy and failure (10, 11). However, despite robust preclinical data, placebo-controlled double-blinded clinical trials targeting cytokines in patients with HF yielded neutral or negative effects on outcomes (12). A plausible explanation for such failures is the lack of temporal and spatial precision to modulate inflammation properly. Such considerations speak to the need for a deeper understanding of how inflammation affects myocardial function.

Neutrophils are the most abundant leukocytes in human blood and are the first responders to infection and injury. During an infection or tissue damage, pathogen-associated molecular patterns (PAMPs) and damage-associated molecular patterns (DAMPs) are sensed by pattern recognition receptors (PPRs) on tissue-resident cells, which produce chemokines such as CXCL1 and CXCL2 to recruit neutrophils (13). In addition, PAMPs and DAMPs may also directly activate neutrophils to induce recruitment and proinflammatory activation (14). During sterile tissue injuries, neutrophils participate in the clearance of cellular debris to restore tissue homeostasis. Neutrophils display a wide range of effector mechanisms such as phagocytosis, cytokine release, matrix protease secretion, and reactive oxygen species (ROS) generation, and the formation of neutrophil extracellular traps (NETs) through a process termed NETosis (15). These neutrophil functions must be tightly regulated, as dysregulated neutrophil activity contributes to persistent inflammation that leads to tissue damage. Regarding cardiac diseases, it has been reported that neutrophils participate in AMI by contributing to thrombosis, removing debris, and eventually orchestrating wound healing (16). However, the role of neutrophils in nonischemic heart diseases and chronic HF remains very poorly understood.

Krüppel-like factors (KLFs) are a family of zinc-finger transcription factors conserved from nematodes to humans. They are essential for a broad spectrum of biological processes, including cell stemness, differentiation, proliferation, malignancy, metabolism, and immunity (17–20). Over the past 2 decades, studies from our group and others have identified KLF2 as a potent repressor of myeloid proinflammatory activation (21, 22). In homeostatic conditions, KLF2 inhibits NF-κB and hypoxia-inducible factor 1 (HIF1) signaling in myeloid cells, thereby maintaining cellular quiescence. Upon proinflammatory stimulation, myeloid KLF2 expression is rapidly diminished to release repression and facilitate myeloid activation. Macrophages and neutrophils that are deficient in KLF2 exhibit more robust proinflammatory activation, rendering hosts resistant to infection but sensitive to systemic inflammation (21). In the current study, we provide multiple lines of evidence to demonstrate that neutrophils are requisite for cardiac pathogenesis in response to chronic nonischemic stress. Further, we use chronic angiotensin II (AngII) infusion as a classic nonischemic cardiac disease model (23) to show a critical role of neutrophil KLF2 in regulating the development and progression of AngII-induced cardiac hypertrophy.

## Results

### HF is associated with reduced KLF2 expression in circulating neutrophils.

**Figure 1:**
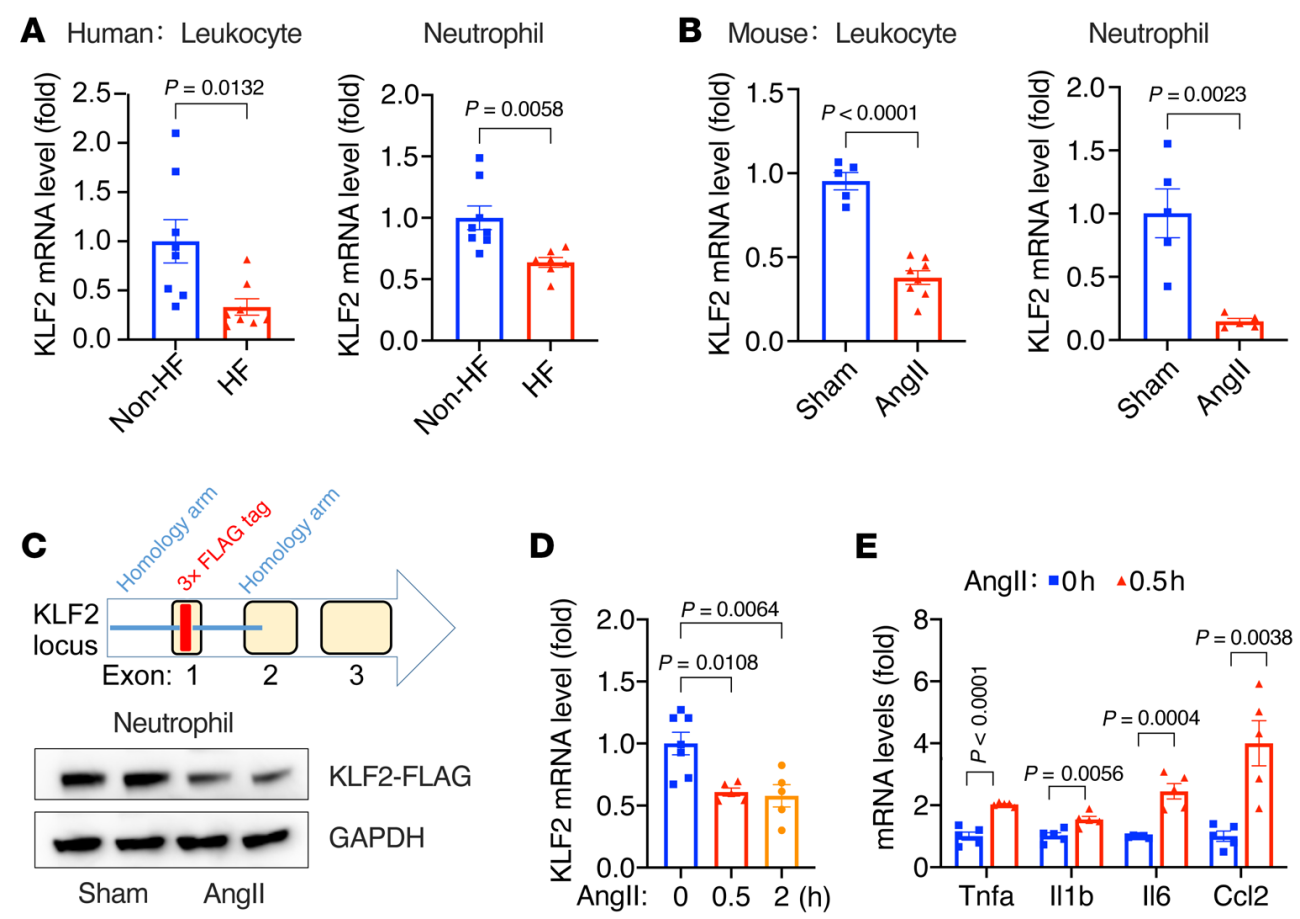
Heart failure is associated with reduced KLF2 expression in circulating leukocytes and neutrophils. (**A**) *Klf2* mRNA expression in human peripheral blood leukocytes (*n =* 8) and neutrophils (*n =* 7–8). HF, patients with heart failure (*n =* 15); non-HF, age-matched patients without heart failure (*n =* 16). (**B**) *Klf2* mRNA expression in peripheral blood leukocytes and neutrophils from WT mice with AngII (1.0 μg/kg/min) or PBS (sham) infusion (*n =* 5–8). (**C**) KLF2-tag mice were generated using the CRISPR/Cas9 method. Protein levels of 3×FLAG-KLF2 in blood neutrophils were detected by M2 anti-FLAG antibody (Sigma-Aldrich, F3165). Each lane represents 1 animal. (**D** and **E**) Mouse bone marrow–derived neutrophils treated with AngII (100 nmol/L) in vitro (*n =* 5–7). *P* values are from 2-tailed, unpaired Student’s *t* test (**A**, **B**, and **E**) or 1-way ANOVA with Tukey’s post hoc test (**D**).

Inflammation is thought to play a critical role in cardiac hypertrophy and failure, but the underlying mechanisms remain elusive (12). Here, we aimed to investigate how immune cells shape cardiac adaptations to pathological stress. Given that KLF2 is a tonic repressor of inflammation, we assessed *Klf2* mRNA levels by quantitative RT-PCR (qPCR) in peripheral blood leukocytes harvested from a cohort of HF patients and non-HF controls (Supplemental Table 1; supplemental material available online with this article; https://doi.org/10.1172/JCI147191DS1). In addition, because the majority (50%–70%) of human circulating leukocytes are neutrophils, we further determined *Klf2* mRNA expression in neutrophils. As shown in Figure 1A, HF is associated with a significant reduction in *Klf2* mRNA levels in both leukocytes and neutrophils. Similar results were observed in mice with 4-week AngII infusion, a classic neurohormonal nonischemic HF model (Figure 1B). However, due to the lack of a high-quality KLF2 antibody, the assessment of KLF2 protein has been a challenge. Therefore, we generated a tagged-KLF2-knockin mouse by inserting a 3×FLAG tag immediately after the ATG codon at the *Klf2* locus using CRISPR/Cas9 gene-editing technology (Figure 1C). This KLF2-tag mouse line allows robust detection of KLF2 protein by commercial high-affinity antibodies against the FLAG epitope, and we confirmed AngII-mediated reduction in KLF2 protein in blood neutrophils in vivo (Figure 1C). These results implicate neutrophil KLF2 in the pathogenesis of cardiac hypertrophy and failure.

Next, we purified bone marrow–derived neutrophils from C57BL/6 WT mice and treated them with AngII in vitro. AngII treatment not only rapidly (within 30 minutes) reduced *Klf2* expression (Figure 1D) but also induced the expression of proinflammatory genes such as *Tnfa*, *Il6*, *Il1b*, and *Ccl2*, indicating AngII-induced proinflammatory activation of neutrophils (Figure 1E and Supplemental Figure 1A). Similar reductions in *Klf2* expression were observed in macrophages, another significant myeloid cell type, but with a much slower (after 24 hours) induction of proinflammatory genes (Supplemental Figure 1B). These cell-based studies suggest that AngII regulates KLF2 expression through cell-autonomous signaling pathways and that AngII can induce proinflammatory activation of myeloid cells. The AngII-mediated direct and rapid signaling in neutrophils further suggests a potential pathogenic role of myeloid KLF2 in cardiac hypertrophy and failure.

### Myeloid KLF2 deficiency enhances AngII-induced cardiac hypertrophy.

To address whether KLF2 can affect HF by mediating myeloid cell quiescence, we employed a mouse model with myeloid cell–specific deletion of *Klf2* via Lyz2-Cre–mediated Cre-lox gene recombination. Previous studies from our group and others have shown the Lyz2-KLF2-KO mice (designated K2KO) exhibit enhanced proinflammatory activation of macrophages and neutrophils compared with the Lyz2-Cre controls (designated Cre) (21, 22).

**Figure 2:**
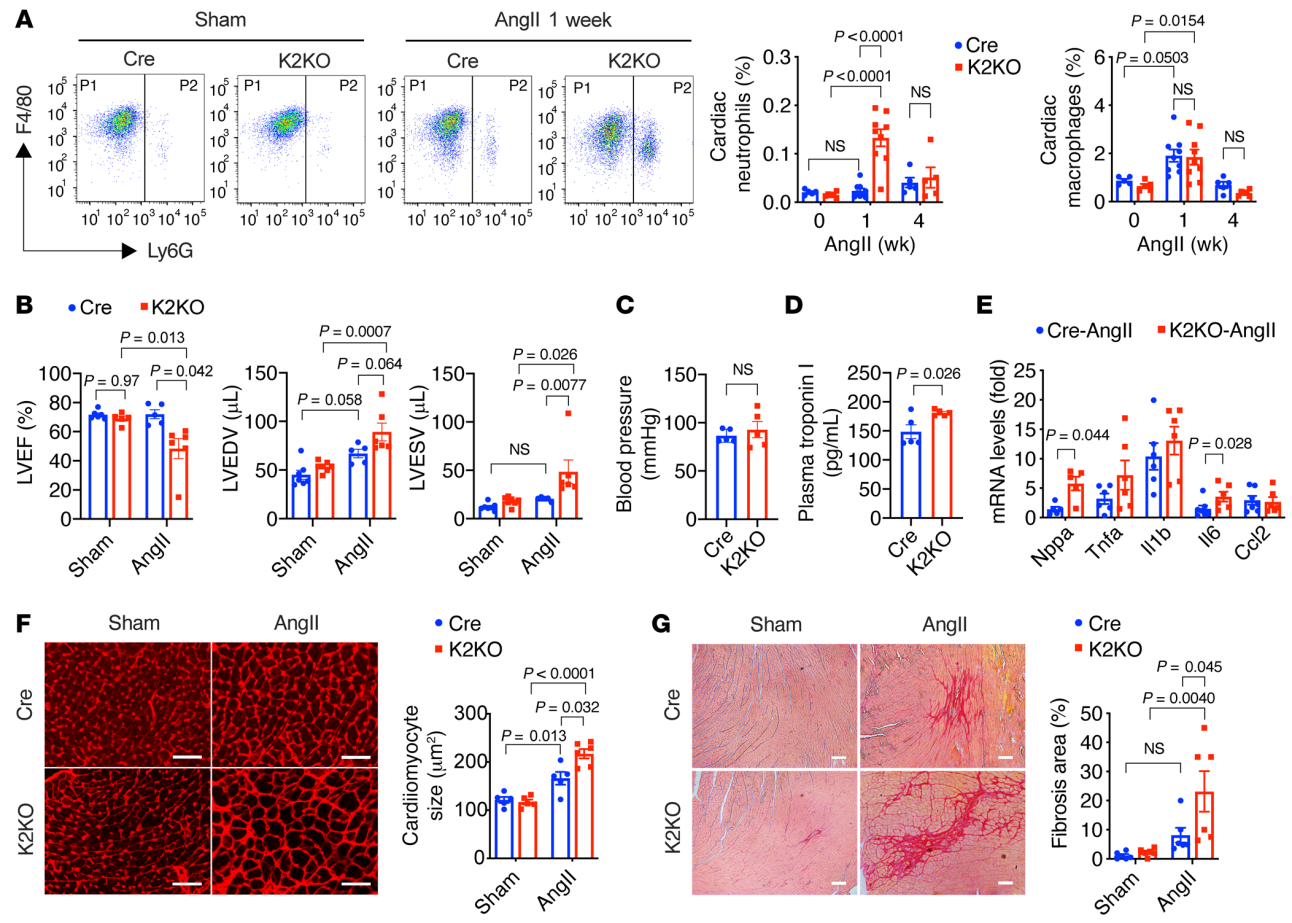
Myeloid KLF2 deficiency enhances AngII-induced cardiac hypertrophy. (**A**) FACS analysis of myeloid cells in the myocardium (*n =* 5–9). P1, CD45^+^CD11b^+^F4/80^+^Ly6G^–^ macrophages; P2, CD45^+^CD11b^+^Ly6G^+^ neutrophils. (**B**) Echocardiography assessments of mouse left ventricular (LV) functions after 4-week infusion (*n =* 5–6). LVEF, left ventricular ejection fraction; LVEDV, LV volume at end of diastole; LVESV, LV volume at end of systole. (**C**) Mean blood pressure measured from the right common carotid artery by invasive hemodynamics (*n =* 5). (**D**) Plasma levels of cardiac troponin (cTnT) after 1 week of AngII infusion (*n =* 5). (**E**) Expression of hypertrophy and inflammation genes in the heart (*n =* 6–7). (**F**) Cardiomyocyte cross-sectional area analysis by Alexa Fluor 594–conjugated wheat germ agglutinin (WGA) staining (*n =* 5–6). (**G**) Myocardial fibrosis analysis by Picrosirius red staining (*n =* 6). *P* values are from 2-way ANOVA with Tukey’s post hoc test (**A**, **B**, **F**, and **G**) or 2-tailed, unpaired Student’s *t* test (**C**–**E**). NS, not significant (*P >* 0.5). Scale bars: 25 μm.

To assess the effects on cardiac hypertrophy and failure, we used the well-established AngII infusion model. In this model, animals develop cardiac hypertrophy and fibrosis at a regular AngII dose of 1.0 μg/kg/min. A higher amount of AngII infusion (2.0 μg/kg/min) can result in cardiac hypertrophy, failure, and death (24, 25). As the first step, we assessed myeloid cell numbers in the heart before and after AngII infusion. Hearts from vehicle-infused (sham: PBS as the vehicle) or AngII-infused (AngII: 1.0 μg/kg/min in PBS) animals were analyzed by flow cytometry. AngII induced significant accumulation of Ly6G^+^ neutrophils in K2KO myocardium, which was increased significantly by 1-week post-AngII infusion and returned to baseline after 4 weeks (Figure 2A). A similar accumulation of cardiac macrophages (CD45^+^CD11b^+^F4/80^+^Ly6G^–^) was observed in both Cre and K2KO groups, without a significant difference between the 2 genotypes. This AngII-induced myeloid response in the heart was more robust at a higher dose of AngII (2.0 μg/kg/min), evidenced by significant accumulation of macrophages and neutrophils in Cre and K2KO hearts, with higher neutrophil numbers in the K2KO group (Supplemental Figure 2, A and B). However, compared with the less than 10% postsurgery mortality with 1.0 μg/kg/min AngII infusion, AngII at 2.0 μg/kg/min resulted in an approximately 50% mortality rate in Cre mice and an approximately 80% mortality rate in K2KO mice within 2 weeks (Supplemental Figure 2C). Therefore, we chose the regular dose of 1.0 μg/kg/min for most in vivo studies.

When subjected to the AngII infusion model for 4 weeks, the K2KO mice developed significantly worse cardiac dysfunction than Cre controls. As revealed by echocardiographic assessments (Figure 2B), Cre and K2KO demonstrated normal cardiac function at baseline. However, after AngII infusion, the K2KO mice exhibited significantly reduced left ventricular ejection fraction (LVEF), increased LV end-diastolic volume (LVEDV), and reduced systolic contraction (increased LV end-systolic volume, LVESV). Of note, this was not due to differences in blood pressure response following AngII infusion (Figure 2C). In addition, plasma levels of cardiac troponin (cTnT) were significantly increased in K2KO mice 1 week after AngII infusion, indicating myocardial injury at the disease onset (Figure 2D). Consistently, K2KO hearts showed increased transcription of cardiac hypertrophy genes (e.g., *Nppa*) and inflammation genes (e.g., *Il6*) (Figure 2E). At the histological level, we observed significant cardiac hypertrophy (enlarged cardiomyocyte cross-sectional area) and fibrosis (collagen-positive area) in the K2KO myocardium (Figure 2, F and G). These AngII-induced pathological changes in the Cre group were less severe than those of the K2KO. Cre mice after 4-week AngII infusion exhibited preserved LVEF, indicating functional cardiac compensation but increased LVEDV, cardiac hypertrophy, and inflammation compared with the sham group (Figure 2, B–G).

Collectively, these data demonstrated that myeloid KLF2 is critical in protecting against AngII-induced cardiac hypertrophy. In response to chronic AngII infusion, KLF2 deficiency likely enhances the proinflammatory activation of myeloid cells, leading to more severe myocardial injury and enhanced cardiac susceptibility to hypertrophy.

### KLF2-deficient neutrophils are critical for AngII-induced cardiac hypertrophy.

Since Lyz2-Cre is operative in both macrophages and neutrophils, we asked which myeloid cell type plays a dominant role in regulating AngII-induced cardiac responses. To address the role of macrophages, we used a Cx3cr1-Cre line to silence *Klf2* exclusively in monocytes and macrophages, thereby maintaining neutrophil KLF2 expression (26). In sharp contrast to the Lyz2-K2KO mice with KLF2 deficiency in both macrophages and neutrophils, the Cx3cr1-Cre–driven KLF2-KO mice (designated Cx3cr1-K2KO) did not manifest severe cardiac hypertrophy in response to 4-week AngII infusion (Supplemental Figure 3). Furthermore, cardiac function was preserved in both Cx3cr1-Cre and Cx3cr1-K2KO groups, while the cardiac hypertrophy was even less in the Cx3cr1-K2KO group, suggesting the KLF2-deficient macrophages are not the primary driver of AngII-induced cardiac dysfunction. Conversely, these data suggest a dominant role of KLF2-deficient neutrophils in HF pathogenesis.

**Figure 3:**
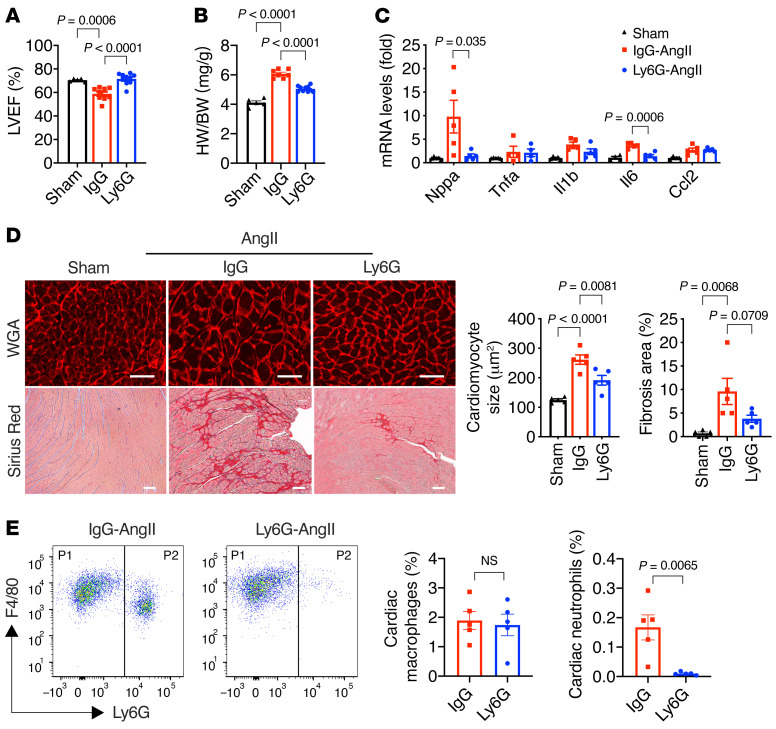
KLF2-deficient neutrophils are critical for AngII-induced cardiac hypertrophy. (**A**) Cardiac function (*n =* 5–10). (**B**) Heart weight (HW) normalized to body weight (BW) (*n =* 5–7). (**C**) Myocardial gene expression (*n =* 4–5). (**D**) Cardiac hypertrophy (WGA staining) and fibrosis (Picrosirius red staining) after 4-week AngII infusion in K2KO mice (*n =* 5). AngII-treated mice were treated with anti-Ly6G antibody (Ly6G) or IgG control antibody (IgG). LVEF, left ventricular ejection fraction. Scale bars: 25 μm. (**E**) FACS analysis of macrophages and neutrophils in K2KO hearts after 1-week AngII infusion and antibody treatments with anti-IgG or anti-Ly6G (*n =* 5). *P* values are from 1-way ANOVA with Tukey’s post hoc test (**A**–**D**) or 2-tailed, unpaired Student’s *t* test (**E**). NS, not significant.

To explore our hypothesis that neutrophils are the major contributors to cardiac phenotype, we treated K2KO mice with anti-Ly6G antibody (clone 1A8) to deplete neutrophils and subsequently subjected them to AngII infusion. Mice receiving normal rat IgG were used as the nondepletion control. Strikingly, neutrophil depletion protected K2KO mice from AngII-induced LV dysfunction (Figure 3A). Furthermore, consistent with preserved cardiac function, neutrophil depletion also reduced myocardial pathologies, including cardiac hypertrophy, fibrosis, and inflammation (Figure 3, B–D). As expected, anti-Ly6G administration essentially eliminated neutrophil infiltration in the myocardium but did not affect cardiac macrophages (Figure 3E), which corroborates the results from the Cx3cr1-K2KO study (Supplemental Figure 3). Collectively, these data demonstrate a critical role of KLF2-deficient neutrophils, rather than macrophages, in the regulation of AngII-induced cardiac hypertrophy.

### NETs are a key mediator of cardiac responses to AngII.

Given the requisite role of neutrophils in AngII-induced cardiac hypertrophy, we sought to understand the underlying pathogenic mechanisms. One well-described mechanism of neutrophil activity is the formation of NETs, sticky web-like structures made of decondensed genomic DNA and decorated with histones and granular proteins such as neutrophil elastase (NE) and myeloperoxidase (MPO). Activated neutrophils release NETs to trap and kill pathogens but can also exacerbate sterile inflammation (15).

**Figure 4:**
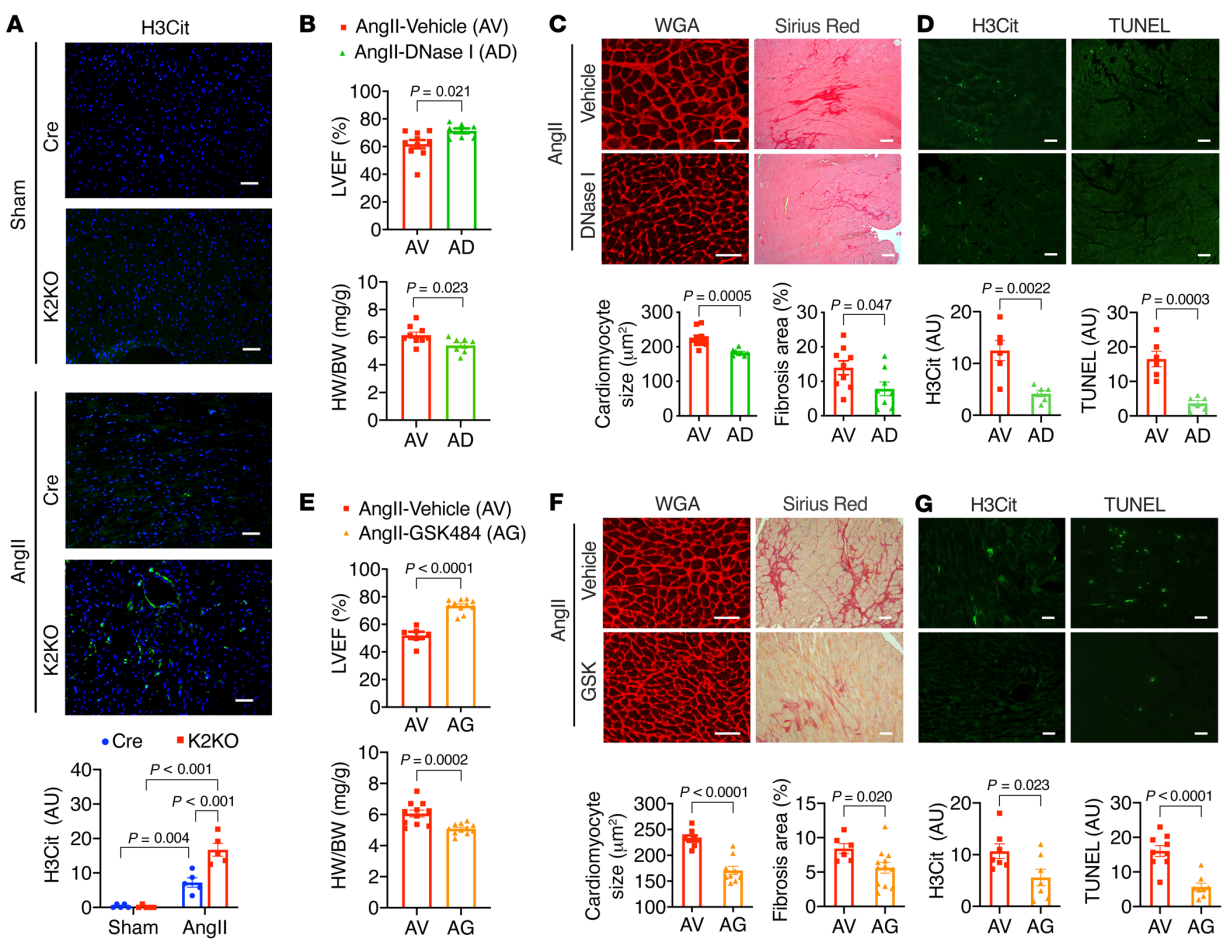
Neutrophil extracellular traps (NETs) as a critical mediator of cardiac responses to AngII. (**A**) Immunostaining of citrullinated histone H3 (H3Cit) from Cre and K2KO hearts (*n =* 5). (**B**–**D**) DNase I administration (*n =* 6–10) and (**E**–**G**) GSK-484 administration (*n =* 6–11) in AngII-infused K2KO mice. (**B **and** E**) LV function and heart weight. (**C **and** F**) Cardiac hypertrophy (WGA staining) and fibrosis (Picrosirius red staining). (**D **and** G**) Intracardiac NET formation (H3Cit immunofluorescence) and cell death (TUNEL immunofluorescence). AngII infusion: 1 week (**A**, **D**, and **G**) or 4 weeks (**B**, **C**, **E**, and **F**). *P* values are from 2-way ANOVA with Tukey’s correction (**A**) or 2-tailed, unpaired Student’s *t* test (**B**–**F**). Scale bars: 25 μm.

As we have shown in Figure 1D, AngII can promote the proinflammatory activation of neutrophils. To investigate the NET formation specifically, we performed fluorescent immunostaining for citrullinated histone H3 (H3Cit), a NET-specific biomarker (27). We observed that 1-week AngII infusion resulted in significantly more H3Cit-positive NETs in the K2KO myocardium than the Cre, with few NETs in untreated hearts from either genotype (Figure 4A and Supplemental Figure 4A). We further confirmed that AngII induced NET formation in vitro with isolated bone marrow neutrophils (Supplemental Figure 4B). Consistent with their proinflammatory nature, K2KO neutrophils formed more NETs than Cre cells. The data from these in vitro and in vivo studies demonstrate that AngII induces NET formation and KLF2 deficiency facilitates this process. Moreover, we observed very few intracardiac NETs after a 4-week AngII infusion (data not shown), indicating a potential pathogenic role of NETs at the disease onset.

To validate the pathogenic role of NETs in vivo, we treated K2KO mice with DNase I (4 mg/kg) during AngII infusion to clear NETs in vivo (28–31). Strikingly, DNase I administration significantly protected K2KO mice from cardiac dysfunction, hypertrophy, and fibrosis (Figure 4, B and C). Furthermore, at the disease onset, DNase I administration cleared intracardiac NETs and attenuated cardiac cell death, as revealed by TUNEL staining (Figure 4D). Subsequently, the myocardial infiltration of neutrophils, but not cardiac macrophages, was also reduced (Supplemental Figure 4C).

In mouse neutrophils, peptidyl arginine deiminase 4 (PAD4) catalyzes the conversion of histone arginine to citrulline in a process termed histone citrullination. This conversion reduces the positive charge on histones, resulting in weakened histone-DNA binding, unwrapping of nucleosomes, and the release of decondensed DNA (32). This process is requisite for NET formation in vivo, as PAD4-KO mice cannot form NETs in response to physiological activators (33). Since DNase I may also degrade extracellular DNA other than that in NETs, we sought to target the NETosis pathway specifically by using GSK-484, a small-molecule PAD4 inhibitor. Our in vivo studies demonstrated that GSK-484 had significant cardioprotective effects similar to DNase I (Figure 4, E–G). By 4 weeks, K2KO mice with GSK-484 treatment were significantly protected from AngII-induced cardiac dysfunction, hypertrophy, and fibrosis (Figure 4, E and F). Furthermore, at the disease onset, GSK-484 treatment eliminated NET formation and cell death in the K2KO myocardium (Figure 4G). Collectively, these data demonstrate that NETs play a critical role in the pathogenesis of AngII-induced cardiac hypertrophy.

### AngII-induced intravascular NET formation causes thrombosis in small vessels and impairs myocardial perfusion.

We next sought to understand how NETs regulate cardiac hypertrophy. We first tested whether NETs or other neutrophil-derived factors could exert direct intercellular crosstalk on cardiomyocytes. Neonatal rat ventricular cardiomyocytes (NRVMs) were isolated and treated with AngII to induce hypertrophy in vitro. Then, to recapitulate the neutrophil-cardiomyocyte crosstalk, we treated NRVMs with neutrophil-conditioned medium (cell-free fraction). As expected, AngII treatment caused hypertrophy of cardiomyocytes, as indicated by the induction of the hypertrophic marker gene *Nppb* (BNP) in NRVMs. Still, no additional effect was observed from adding neutrophil-conditioned medium (Supplemental Figure 5, A and B). Further, similar in vitro studies with cardiac fibroblasts did not reveal any detectable effects of neutrophil-conditioned medium to enhance the expression of fibrosis genes (Supplemental Figure 5C). These data suggest that the prohypertrophic, profibrotic results of neutrophils observed in vivo are more complicated than direct paracrine crosstalk and may involve other cells or systems in the myocardium.

The location of a cell in tissues can provide clues to function. Therefore, we carefully mapped the localization of neutrophils and NETs in the myocardium. When we overlaid the immunofluorescence of neutrophil marker Ly6G and endothelial marker CD31, we found that most neutrophils in the myocardium appeared to localize inside small vessels or on large vessel walls, with few in the intramuscular space (Supplemental Figure 6A). A similar localization pattern was observed with H3Cit and CD31, indicating that most NETs were formed inside small vessels (Supplemental Figure 6B). This observation raised the possibility that neutrophils and NETs contribute to thrombosis and resultant tissue ischemia in a process termed immunothrombosis to cause myocardial injury (27, 34). Indeed, NETs promote thrombosis in both acute infection (i.e., sepsis, COVID-19) and chronic inflammation (i.e., deep vein thrombosis; refs. 27, 31, 35). And NETs can regulate coagulation at multiple levels: activation of intrinsic coagulation factors (i.e., factor XII, XI, X), enrichment in extrinsic coagulation initiator tissue factor, proteolytic degradation of tissue factor pathway inhibitor (TFPI), facilitation of platelet adhesion, and interaction with fibronectin and von Willebrand factor (vWF) for thrombus propagation (34, 36, 37). As such, we hypothesized that NETs in small vessels might cause sporadic thrombosis and subsequently impair blood perfusion of the myocardium.

**Figure 5:**
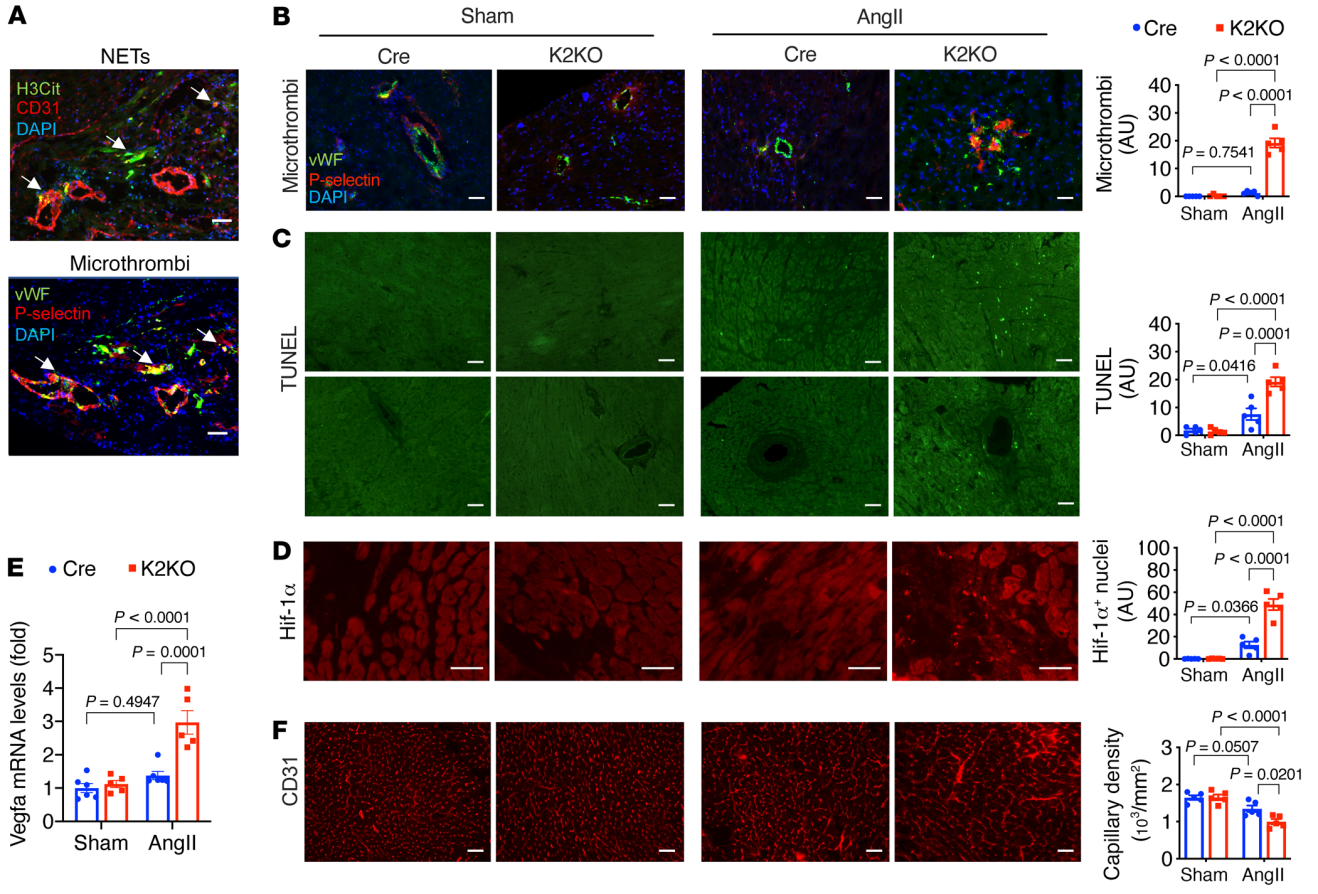
AngII-induced NET formation triggers microthrombosis and myocardial injury. (**A **and** B**) Immunostaining of H3Cit, vWF, P-selectin, and CD31. (**C**) TUNEL staining to assess cell death. Upper: Intramuscular regions. Lower: Perivascular regions. (**D**) Immunostaining of HIF1α protein. HIF1α-positive nuclei were counted. (**E**) Myocardial expression of *Vegfa* mRNA. (**F**) Myocardial capillary density assessed by CD31 immunostaining. AngII infusion: 1 week (**A**–**D**) or 4 weeks (**E** and **F**). *P* values are from 2-way ANOVA with Tukey’s correction (**B**–**F**). Representative images from 5 to 6 mice in each group. Scale bars: 25 μm.

In the AngII-treated myocardium, we found platelet-rich microthrombi (stained for P-selectin) localized inside small vessels (stained for vWF), a localization pattern similar to the NETs (stained for H3Cit; Figure 5A and Supplemental Figure 6C). Significant microthrombi were observed in the K2KO myocardium, but they were scarce in the Cre group (Figure 5B and Supplemental Figure 6D). As a consequence of thrombosis, such affected regions in the K2KO myocardium would likely have suffered from ischemic stress. Indeed, TUNEL staining revealed significant cell death, mainly involving cardiomyocytes and a small number of vascular cells (Figure 5C). While the death of cardiomyocytes could account for the increased plasma levels of cTnT (Figure 2B), the death of vascular cells suggests NETs may trigger vascular injury secondary to thrombosis or through a direct mechanism such as ROS. In support of the thrombotic hypothesis, myocardial ischemia was evident by the increased nuclear accumulation of HIF1α protein (Figure 5D) and induction of *Vegfa* expression (Figure 5E). Surprisingly, despite higher expression of *Vegfa*, the myocardial capillary density was significantly reduced in the AngII-infused K2KO heart compared with Cre (Figure 5F), which could impair blood perfusion and further enhance ischemia. Thus, the reduced capillary density in AngII-infused K2KO hearts could be due to attenuated angiogenesis and/or enhanced capillary rarefaction secondary to microvascular thrombosis. Our data on myocardial *Vegfa* mRNA levels do not support an angiogenesis defect (Figure 5E). Still, the data on TUNEL-positive vascular cells support capillary rarefaction (Figure 5C).

Nevertheless, these data support our hypothesis that AngII induces intravascular NET formation, leading to sporadic thrombosis and hypoxia in the myocardium. Consistent with the cardiac phenotypes, AngII-induced pathological changes in thrombosis, ischemia, cell death, and capillary density were most significant in the K2KO group and undetectable in sham groups. In contrast, K2KO animals depleted of neutrophils (by anti-Ly6G) or NETs (by DNase I and GSK-484) were dramatically protected not only from AngII-induced cardiac dysfunction (Figures 3 and 4) but also from proximal events such as intracardiac NETs, thrombosis, cell death, and capillary rarefaction (Supplemental Figure 7).

**Figure 6:**
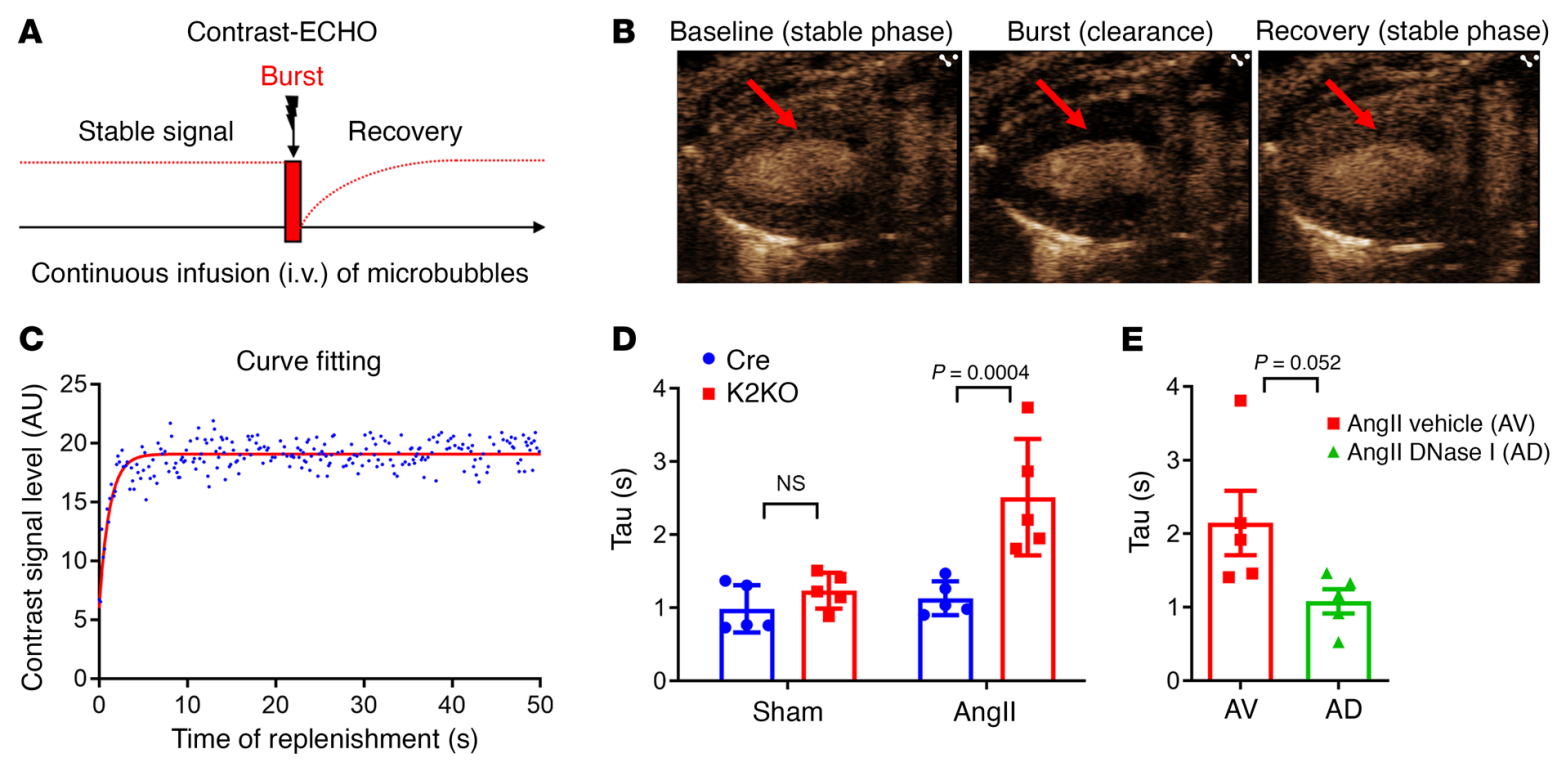
AngII infusion impairs microcirculation in the K2KO myocardium. (**A**) The experimental design of contrast-ECHO showing 3 phases of the contrast signal: basal stable level, clearance by a burst of high-energy ultrasound beam, and recovery. The rate of contrast signal recovery is correlated with the microcirculatory blood flow rate. (**B**) Representative contrast-ECHO images showing baseline, burst, and complete recovery phases. Arrows indicate LV wall. (**C**) Representative data analysis showing cure fitting of a 1-phase exponential decay curve. The recovery rate (blood flow rate) can be estimated by time constant (Tau) of the curve. A higher Tau value indicates slower blood flow. (**D**) Contrast-ECHO data from Cre and K2KO mice before and after 4-week AngII infusion (*n =* 5). *P*_(interaction)_ = 0.0143 by 2-way ANOVA. *P* value shown is from Tukey’s post hoc test. NS, not significant. (**E**) The effect of DNase I administration on myocardial microcirculation assessed by contrast-ECHO (*n =* 5). *P* value from 2-tailed, unpaired Student’s *t* test.

To determine if myocardial perfusion is indeed affected by the AngII-NET-immunothrombosis mechanism hypothesized above, we assessed myocardial microcirculation using quantitative contrast echocardiography (contrast-ECHO, as illustrated in Figure 6A). Briefly, mice were intravenously infused with a microbubble solution (contrast agent) at a constant flow rate to establish a stable concentration of microbubbles in the myocardium. Then, a transient high-energy ultrasonic beam was applied at the precise time to destruct the microbubbles in the myocardium (burst). Subsequently, intratissue microbubbles were replenished at a rate proportional to the blood flow rate of the myocardial microcirculation. Under continuous echocardiographic monitoring, the stable level of microbubbles in the myocardium (baseline, stable phase before the burst), the “zero” level after destruction (clearance of microbubbles by the burst), and the recovery to stable level (replenishment after the burst) can be recorded in real time by echocardiography (Figure 6B). Time-stamped contrast-ECHO images then can be calculated and fit into a 1-phase decay exponential curve to estimate the myocardial blood flow rate (Figure 6C). The time constant (Tau) from the fitting curve was used as an indicator of flow rate. A higher Tau value indicates a longer recovery time; that is, microbubbles from blood need a longer time period to replenish the myocardium, indicating a slower blood flow rate. As shown in Figure 6D, there was little difference between the Cre and K2KO mice at baseline. However, AngII infusion induced a profound change in myocardial perfusion in the K2KO mice, with a Tau value approximately 2-fold larger than that of the Cre (2.51 ± 0.36 vs. 1.13 ± 0.10), indicating impaired microcirculation in the K2KO myocardium in response to AngII infusion. In addition, such impairment in myocardial microcirculation was largely restored by DNase I administration (Figure 6E), supporting a causative role of NETs in this process.

### The anticoagulant heparin ameliorates AngII-induced cardiac dysfunction.

**Figure 7:**
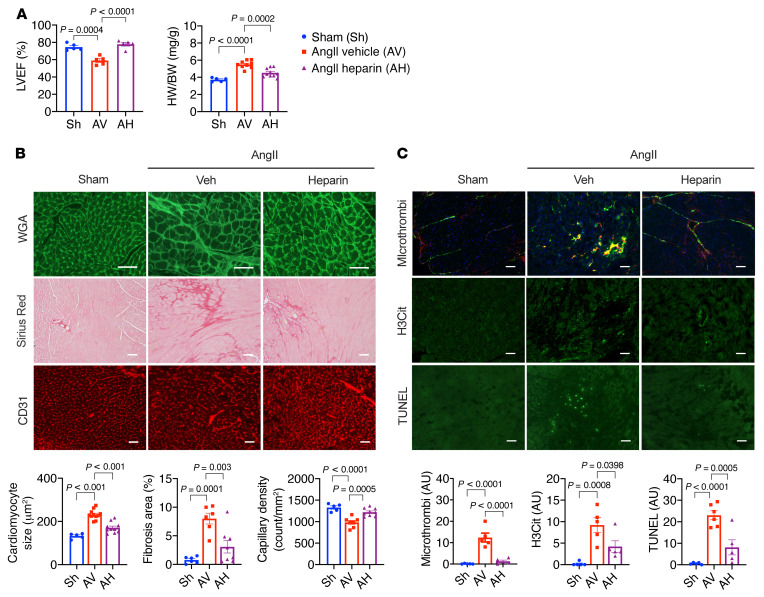
Heparin administration ameliorates AngII-induced cardiac dysfunction in K2KO hearts. (**A**) Cardiac function and hypertrophy (*n =* 5–10). (**B**) Myocardial hypertrophy (WGA–Alexa Fluor 488 staining), fibrosis (Picrosirius red staining), and capillary density (CD31 immunofluorescence). *n =* 5–11. (**C**) Intracardiac microthrombosis (vWF/P-selectin immunofluorescence), NET formation (H3Cit immunofluorescence), and cell death (TUNEL immunofluorescence). Infusion: 4 weeks (**A** and **B**) or 1 week (**C**). *P* values are from 1-way ANOVA with Tukey’s post hoc test. Representative images from an individual animal (*n =* 5–11 in each group). Scale bars: 25 μm.

Next, we investigated whether anticoagulant therapies can be cardioprotective in this model. For this purpose, we used heparin, one of the most commonly used anticoagulants in the clinical setting (38). K2KO mice were subjected to AngII infusion (1.0 μg/kg/min) with or without the concurrent infusion of heparin (1.0 U/kg/min), and cardiac function and pathologies were subsequently assessed (Figure 7). By the end of 4-week AngII infusion, heparin treatment preserved cardiac function, reduced cardiac hypertrophy, attenuated myocardial fibrosis, and improved angiogenesis in the myocardium (Figure 7, A and B). Mechanistically, heparin infusion significantly attenuated intracardiac thrombosis, NET formation, and cell death at the disease onset (Figure 7C).

Collectively, these data demonstrate that in the absence of KLF2 in neutrophils, AngII-induced intravascular NET formation triggers thrombosis, particularly in small vessels with a narrow lumen (prone to blockade) and low flow rate (prone to clot), leading to reduced or absent perfusion in affected myocardial regions. Although sporadic, NET-triggered thrombosis may result in localized myocardial ischemia, cell death, and capillary rarefaction, which are known pathogenic factors for cardiac hypertrophy and HF. As such, therapies that target neutrophils (Figure 3), NETs (Figure 4), or thrombosis (Figure 7) can be beneficial to the heart.

### The AngII/NET axis is operative in WT mice.

Using the myeloid KLF2–deficient (K2KO) mice as a proinflammatory genetic model, we demonstrated that the neutrophil/KLF2/NET axis is critical for regulating cardiac hypertrophy. Furthermore, given that the reduction in KLF2 expression in neutrophils is observed from both clinical HF patients and experimental HF mice (Figure 1) and that KLF2 reduction is known to enhance proinflammatory myeloid cell activation (39), we sought to validate whether this pathogenic pathway is operative in WT animals, where the KLF2 gene is not deleted, but rather its expression is reduced in the setting of disease.

We first used an anti-Ly6G antibody to deplete neutrophils in WT mice to see whether it protects the heart from hypertrophy. It is well known that C57BL/6 mice do not develop severe cardiac hypertrophy in response to a regular dose of AngII (1.0 μg/kg/min). Therefore, we used a higher dose of AngII (2.0 μg/kg/min) to probe the protective roles of neutrophil depletion. At this high dose, AngII also induced a high mortality rate (Supplemental Figure 2C), likely due to aortic dissection (data not shown). At 4 weeks after AngII infusion, the surviving mice showed significant improvement in cardiac functions and hypertrophy in the anti-Ly6G antibody–treated (neutropenia) group compared with the normal IgG antibody–treated (control) groups (Supplemental Figure 8), indicating a pathogenic role of WT neutrophils that is similar to the KLF2-null condition.

**Figure 8:**
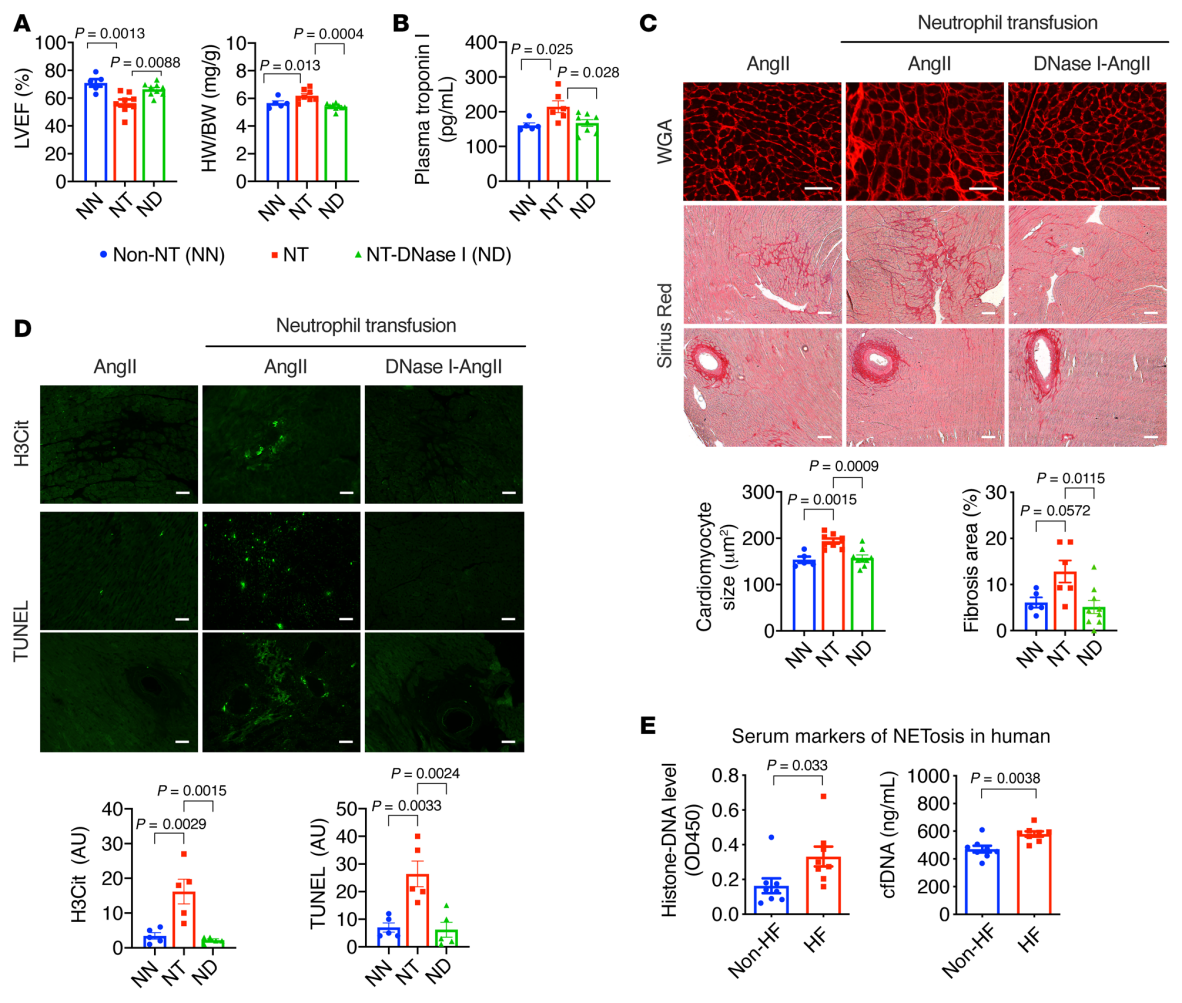
Neutrophilia by adoptive neutrophil transfusion accelerates AngII-induced cardiac hypertrophy. (**A**) LV function and hypertrophy. Non-NT, no neutrophil transfusion; NT, neutrophil transfusion; NT-DN, neutrophil transfusion plus DNase I treatment. All groups received a 4-week AngII infusion (*n =* 5–9). (**B**) Plasma cardiac troponin I (cTnT) levels after 1-week AngII infusion (*n =* 5–8). (**C**) Myocardial hypertrophy (WGA staining) and fibrosis (Picrosirius red staining). (**D**) Intracardiac NETs (H3Cit) and cell death (TUNEL). (**E**) Histone-associated DNA fragments and cell-free DNA (cfDNA) in the plasma of HF patients and non-HF controls (*n =* 8). *P* values are from 1-way ANOVA with Tukey’s correction (**A**–**D**) or 2-tailed, unpaired Student’s *t* test (**E**). Representative images from an individual animal (*n =* 5–9 in each group). Scale bars: 25 μm.

Next, we assessed whether neutrophilia established by adoptive transfusion could augment cardiac dysfunction in WT mice. During the 4-week AngII infusion, 5 million neutrophils isolated from WT donors’ bone marrow were transfused every week via intravenous injection. It has been reported that transfusion of 5 million neutrophils is well tolerated by normal mice without severe adverse effects (30), resulting in acute neutrophilia within 2 hours and slightly increased leukocyte counts after 24 hours, but no change in platelet counts (Supplemental Figure 9). In mice with AngII infusion, neutrophil transfusion significantly accelerated cardiac hypertrophy, resulting in reduced LV contractility and enlarged hearts (Figure 8A). Plasma cTnT levels were also elevated in the transfusion group, indicating more severe myocardial injury (Figure 8B). Pathological analyses revealed more severe cardiomyocyte hypertrophy, myocardial fibrosis, cell death, and NET formation in neutrophil-transfused hearts (Figure 8, C and D). Consistent with the pathogenic roles of NETs, simultaneous administration of DNase I with neutrophil transfusion almost completely blocked the adverse effects (Figure 8, A–D). These data demonstrate that enhanced neutrophil function accelerates AngII-induced cardiac hypertrophy, likely through the NET/thrombosis axis.

To determine the clinical relevance of the neutrophil/KLF2/NET axis, we analyzed plasma samples from HF patients and non-HF control subjects for NET-related biomarkers, including histone-associated DNA fragments and cell-free DNA (cfDNA). Compared with the non-HF group, we detected significantly higher levels of histone-associated DNA fragments and cfDNA in the HF group (Figure 8E), suggesting increased NET formation in HF patients. Given that HF patients often have heightened activity of the renin-angiotensin system (RAS) and reduced neutrophil KLF2 levels, these results suggest that the AngII/neutrophil/KLF2/NET axis may contribute to human HF.

### KLF2 is critical for the transcriptional regulation of neutrophils in cardiac hypertrophy.

To understand mechanistically how changes in the neutrophil transcriptome governed by neutrophil KLF2 regulates cardiac hypertrophy, we sorted the Ly6G^+^ neutrophils from the myocardium and performed RNA-Seq studies. We picked the 1-week post-AngII-infusion time point for neutrophil isolation because it is both the peak of neutrophil infiltration and the onset of heart disease. Since there were very few neutrophils in the myocardium at baseline (Figure 2A), we only focused on the AngII-infused groups. In total, approximately 5000 neutrophils were included in each sample (Cre vs. K2KO, *n =* 4) for RNA extraction. RNA-Seq was performed using the low-RNA-input protocol. We identified 1740 differentially expressed genes (DEGs) with a significant *P* value of less than 0.05 and 2-fold change in expression levels (Supplemental Figure 10A).

**Figure 9:**
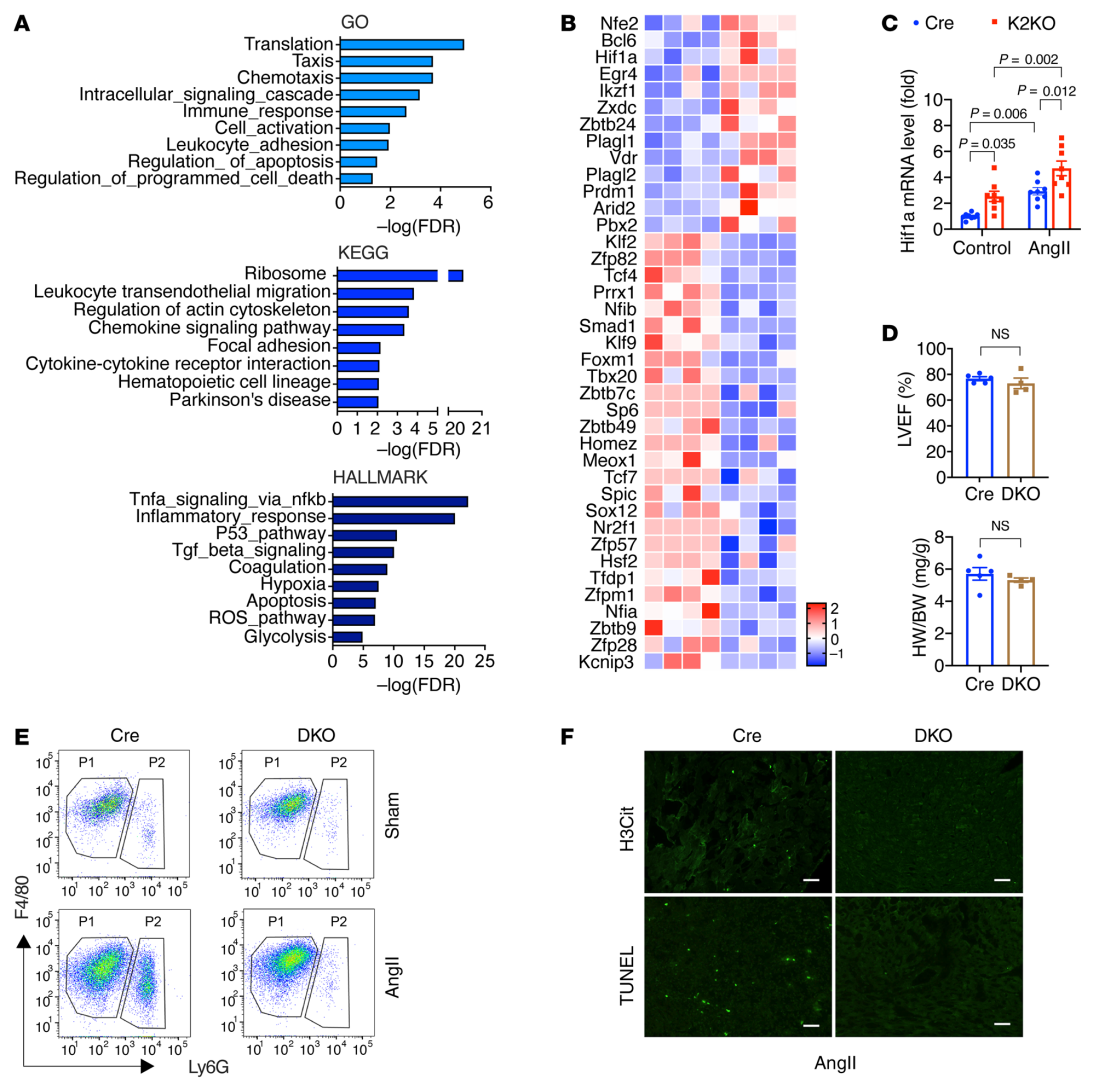
Transcriptomic studies identify KLF2 as a nodal regulator in neutrophils. (**A **and** B**) Pathway enrichment analysis and heatmap of all transcription factors in neutrophil DEGs (Cre-AngII vs. K2KO-AngII). RNA-Seq studies included 4 animals in each group. (**C**) *Hif1a* expression in neutrophils (*n =* 6). Treatment: AngII (100 nmol/L) for 0.5 hours in vitro. *P* values are from 2-way ANOVA with Tukey’s correction. (**D**–**F**) Lyz2-Cre (Cre) vs. Lyz2-Cre-KLF2-HIF1α double-knockout (DKO) mice (*n =* 4–5). (**D**) Cardiac function and hypertrophy. NS indicates not significant by 2-tailed, unpaired Student’s *t* test. (**E**) FACS analysis of cardiac myeloid cells. (**F**) Intracardiac NET formation (H3Cit) and cell death (TUNEL). Scale bars: 25 μm. Representative FACS and immunofluorescence images from an individual animal (*n =* 5). AngII infusion: 4 weeks (**D**) or 1 week (**E** and **F**).

The gene ontology (GO) and pathway enrichment analyses with DEGs (Figure 9A) in GO and KEGG pathways were enriched in protein translation (ribosome function), cytokine/chemokine signaling, inflammation, leukocyte migration, and adhesion, which strongly support the proinflammatory phenotypes we observed with K2KO neutrophils in vivo and in vitro. Furthermore, the GO terms on apoptosis and programmed cell death may be associated with NETosis, a unique form of programmed cell death. Hallmark pathway analysis revealed 30 Hallmark pathways (*P <* 0.00001), including inflammation-related pathways (TNF-α, p53, ROS, TGF-β, apoptosis), metabolic pathways (glycolysis, hypoxia), and coagulation pathway (Figure 9A and Supplemental Figure 10B). Together, findings from these transcriptomic analyses support a crucial role of KLF2 in neutrophil biology in response to AngII.

We next investigated the transcriptional network that KLF2 may control in neutrophils in response to AngII. Among the 1740 DEGs, we identified 39 transcription factor genes that were changed over 2-fold (|FC| > 2), including 26 downregulated and 13 upregulated transcription factor genes (Figure 9B). As expected, *Klf2* was found to be the most significantly (*P <* 0.00005) downregulated transcription factor gene in K2KO neutrophils. Conversely, the top 3 most significantly upregulated transcription factor genes were *Nfe2*, *Bcl6*, and *Hif1a*. NFE2 (nuclear factor erythroid 2) is associated with myeloproliferative neoplasms and polycythemic disorders (40). BCL6 (B cell lymphoma 6) has been reported to regulate apoptosis in neutrophils, which may be involved in NETosis (41). Notably, HIF1α is an oxygen-sensing subunit of the heterodimeric transcription factor HIF1, a master regulator of myeloid cells (42). HIF1α transcriptionally regulates glycolysis, the dominant metabolic pathway that supports ATP synthesis in proinflammatory myeloid cells and many cytokine genes such as that encoding IL-1β (*Il1b*) (43). HIF1α also induces the expression of NFE2 (44). Therefore, we focused on investing the potential crosstalk between KLF2 and HIF1α signaling pathways.

KLF2 has been reported to negatively regulate HIF1α signaling in myeloid cells and other cell types (21, 45). As revealed by RNA-Seq, KLF2 deficiency augmented HIF1α (*Hif1a*) expression in neutrophils in response to AngII (Figure 9B). This response was recapitulated in vitro using isolated mouse bone marrow neutrophils. The results showed that KLF2-deficient neutrophils expressed higher levels of *Hif1a* mRNA both at baseline and after AngII treatment (Figure 9C). To confirm a KLF2/HIF1α axis in vivo, we generated Lyz2-Cre–driven, myeloid-specific, KLF2-HIF1α double-knockout mice (designated DKO) and subjected them to the AngII infusion model. At baseline, the DKO mice are grossly normal without any visible defects. However, after a 4-week AngII infusion, the cardiac function of DKO mice was well preserved to a level comparable to Cre mice (Figure 9D). Furthermore, AngII-induced myocardial neutrophil infiltration, NET formation, and cell death were primarily abolished in the DKO myocardium (Figure 9, E and F). These data demonstrate that, in response to AngII, compound deficiency of both KLF2 and HIF1α restrains neutrophil proinflammatory activation and preserves cardiac function. This further suggests that hyperactivation of HIF1α signaling resulting from neutrophil KLF2 deficiency exacerbates inflammation and worsens cardiac hypertrophy. Collectively, these transcriptomic and genetic studies indicated that the KLF2/HIF1α axis is critical for AngII-induced neutrophil activation and cardiac hypertrophy, likely through the regulation of NETosis and resultant thrombosis in small vessels of the myocardium.

### Neutrophils orchestrate the myocardial inflammatory responses.

Neutrophils are the first responders to injury or inflammatory stimuli, capable of forming complex crosstalk with other cells, including immune cells and mural cells. To understand how neutrophils orchestrate the myocardial responses to AngII, we performed single-cell RNA-Seq (scRNA-Seq) with live non-cardiomyocytes sorted from the Cre and K2KO hearts.

**Figure 10:**
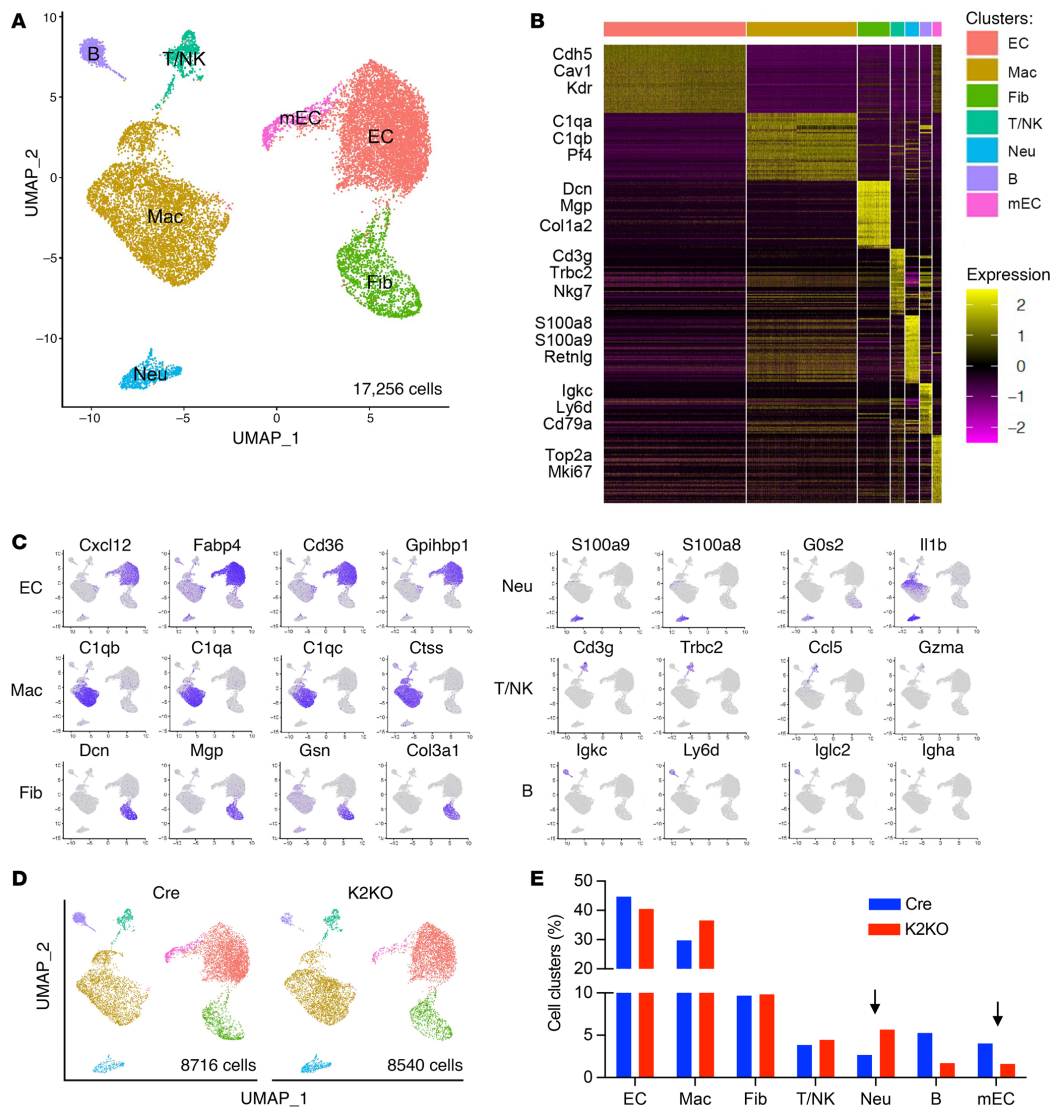
Single-cell RNA-Seq study identifies the major non-cardiomyocyte cell types that regulate cardiac dysfunction. (**A**) UMAP and unsupervised clustering analysis using Seurat pipeline identified 7 distinct cell populations from a total of 17,256 cells. EC, endothelial cell; Mac, macrophage; Fib, fibroblast; T/NK, T cell and NK cell; Neu, neutrophil; B, B cell; mEC, mitotic endothelial cell. (**B**) Heatmap of top 50 marker genes for each cluster. Selected cell-type-specific markers labeled. (**C**) Feature plots depicting gene expression on UMAP. (**D**) UMAP of 8716 Cre cells and 8540 K2KO cells showing 7 cell populations. (**E**) Percentage of each cell cluster in Cre and K2KO groups. Differences in neutrophils and mitotic endothelial cells are noted. Cells isolated from 3 mice in each group were pooled before FACS isolation. Two pooled samples (Cre vs. K2KO) were single-cell captured and sequenced.

We subjected Cre and K2KO mice to 1-week AngII infusion to capture the pathogenic changes at the disease onset. After enzymatically dispersing cardiac tissue, cells were sorted using flow cytometry. Dead cells were excluded by live/dead dye, and live cells were sorted to enrich CD45^+^ cells and CD31^+^ cells. Single-cell capturing and library preparation was performed using a 10× Genomics Chromium Single Cell 3′ GEM, Library and Gel Bead Kit v3, followed by next-generation sequencing using the Illumina platform. After preprocessing raw sequencing data and the quality control of cell barcodes and unique molecular identifiers (UMIs), 8716 cells from the Cre group and 8540 cells from the K2KO group were subject to downstream bioinformatics analysis. Uniform manifold approximation and projection for dimension reduction (UMAP) and unsupervised clustering analysis using Seurat pipeline identified 7 distinct cell populations from the total 17,256 cells (Figure 10A). Gene expression patterns of established canonical markers of various immune cell lineages (*C1qa*, *C1qb*, *S100a8*, *S100a9*, *Cd3g*, *Ly6d*, *Cd79a*, etc.), endothelial cells (ECs) (*Cdh5*, *Cav1*, *Kdr*, etc.), and fibroblasts (*Dcn*, *Mgp*, *Col1a2*, etc.) allowed the assignment of putative biological identities to each cluster, namely neutrophils, macrophages, B cells, T/NK cells, conventional ECs, mitotic ECs, and fibroblasts. Cell-type-specific markers are shown as a heatmap of top 50 marker genes for each cluster (Figure 10B), feature plots depicting gene expression on UMAP (Figure 10C), and a dot plot for top 4 marker genes for each cluster (Supplemental Figure 11A). Between the Cre and K2KO groups, it appeared that the neutrophil cluster was increased (2.67% in Cre vs. 5.62% in K2KO). In comparison, the mitotic EC cluster was reduced (4.04% in Cre vs. 1.59% in K2KO) in the K2KO group (Figure 10, D and E), faithfully recapitulating the excessive neutrophil infiltration (Figure 2A) and capillary rarefaction phenotypes (Figure 5F) at the mRNA level.

**Figure 11:**
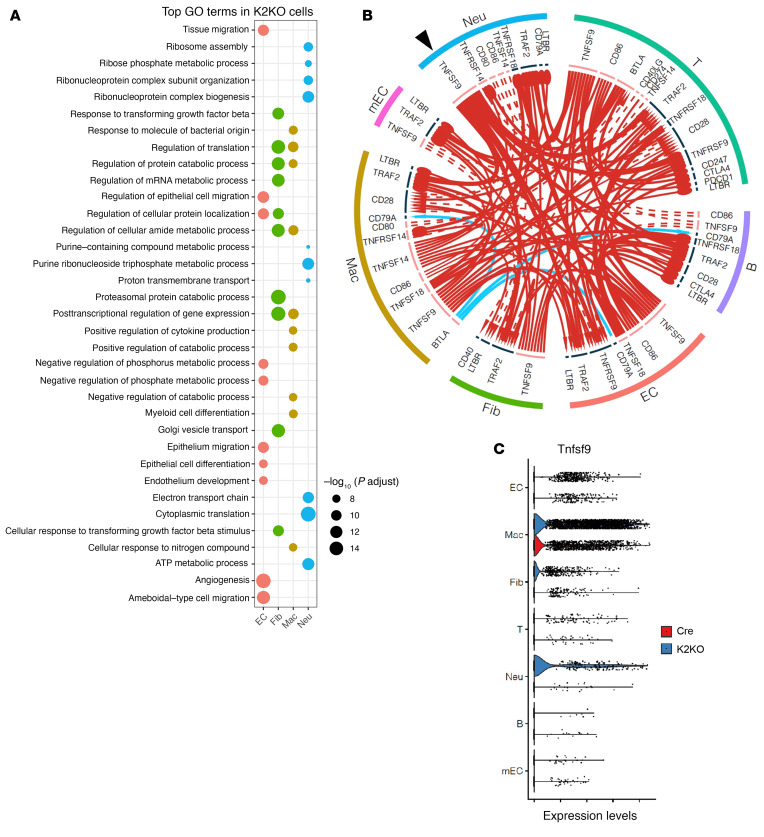
Neutrophils orchestrate myocardial inflammation and adaptation to AngII stress. (**A**) Gene ontology (GO) analyses with K2KO DEGs from 4 major cell types: neutrophils, macrophages, endothelial cells, and fibroblasts; showing top 10 biological process (BP) GO terms according to adjusted *P* values (p.adjust). (**B**) Cell-cell interactome analysis of all significant 7 cell types based on the ligand-receptor communication. Arrows: red = upregulated, blue = downregulated; arrowhead = receptor level changed, circle head = receptor level NOT changed; solid line = ligand level changed, dotted line = ligand level NOT changed; line thickness and head size represent relative fold change values. (**C**) *Tnfsf9* mRNA expression levels in all significant 7 cell types are shown as violin plot.

Next, we performed GO analysis with DEGs (K2KO vs. Cre, *P*_adj_ < 0.05), particularly in the significant 4 cell types: neutrophils, macrophages, ECs, and cardiac fibroblasts (Supplemental Figure 11B). Upregulated DEGs in K2KO neutrophils were enriched in GO terms related to protein synthesis (ribosome assembly, cytoplasmic translation), energy metabolism (ATP metabolic process and electron transfer chain), purine ribonucleotide and purine-containing compound metabolism, ribose phosphate metabolic process, and ribonucleoprotein complex–related processes, indicating a robust activation status of the K2KO neutrophils (Figure 11A). In particular, GO terms related to ribonucleoprotein complexes and energy metabolism likely supported the enhanced NETosis observed in K2KO neutrophils (46). Furthermore, purine ribonucleotides and purine-containing compounds are classic biomarkers of neutrophil activation and mediators of EC dysfunction (47, 48). In contrast, upregulated DEGs in Cre neutrophils were enriched in GO terms related to classic immune functions, including neutrophil activation, leukocyte activation, chemotaxis, leukocyte migration, cytokine-mediated signaling pathway, responses to infection (viral and other organisms), and wounding (Supplemental Figure 12).

K2KO macrophage showed enrichment in GO terms “myeloid cell differentiation,” “positive regulation of cytokine production,” and “response to molecule of bacteria origin,” indicating proinflammatory activation of cardiac macrophages during cardiac hypertrophy (Figure 11A). Cre macrophages showed enrichment in GO terms related to antigen presentation, mitochondrial function, RNA splicing, protein folding and catabolic processes, and ribose/nucleoside metabolism (Supplemental Figure 12). We have shown that AngII induced similar levels of macrophage accumulation in Cre and K2KO myocardium, but the GO analysis demonstrated vast differences in their function.

Consistent with the myocardial fibrosis phenotype, K2KO fibroblast GO terms were enriched in TGF-β signaling, gene transcription, and translation-related related processes, proteasomal protein degradation, and Golgi vesicle transport processes. At the same time, there was minimal GO enrichment in Cre fibroblasts (Figure 11A and Supplemental Figure 12). In K2KO ECs, GO terms were enriched in angiogenesis, EC development and migration, and regulation of cellular protein localization. In contrast, Cre ECs showed enrichment in “cytoplasmic translation,” “establishment of endothelial barrier,” ATP synthesis–related processes, protein folding, etc. (Figure 11A and Supplemental Figure 12). Thus, the EC GO analysis suggests heightened EC activation and impaired endothelial barrier function in the K2KO myocardium, underscoring myocardial perfusion and capillary density impairments.

Finally, to gain insight into the complex myocardial responses orchestrated by neutrophils, particularly the KLF2-deficient neutrophils, we performed cell-cell interactome analysis, comparing Cre and K2KO groups. The most significant changes (i.e., the biggest fold changes) of ligand-receptor communication between different cell types revealed a complex immune checkpoint network among all major non-cardiomyocyte cell types (Figure 11B). Interestingly, among genes related to an immune checkpoint, TNF superfamily member 9 (*Tnfsf9*, also known as 4-1BB ligand) is one of the most significantly upregulated ligands both in neutrophils and macrophages of the K2KO group and transacts signals with multiple cell types. The cluster-specific expression of *Tnfsf9* is shown separately in Figure 11C. It has been reported that TNF signaling accelerates thrombosis and fibrosis in vivo (49), and TNFSF9 is implicated in lung inflammation and fibrosis (50). Our results thus suggest the potential role of TNFSF9 in HF. Collectively, these scRNA-Seq data demonstrate that neutrophils are the crucial regulator orchestrating myocardial inflammation because alterations in neutrophil function (due to KLF2 deficiency) significantly reshape the immune responses from all significant non-cardiomyocyte cell types in the myocardium.

## Discussion

**Figure 12:**
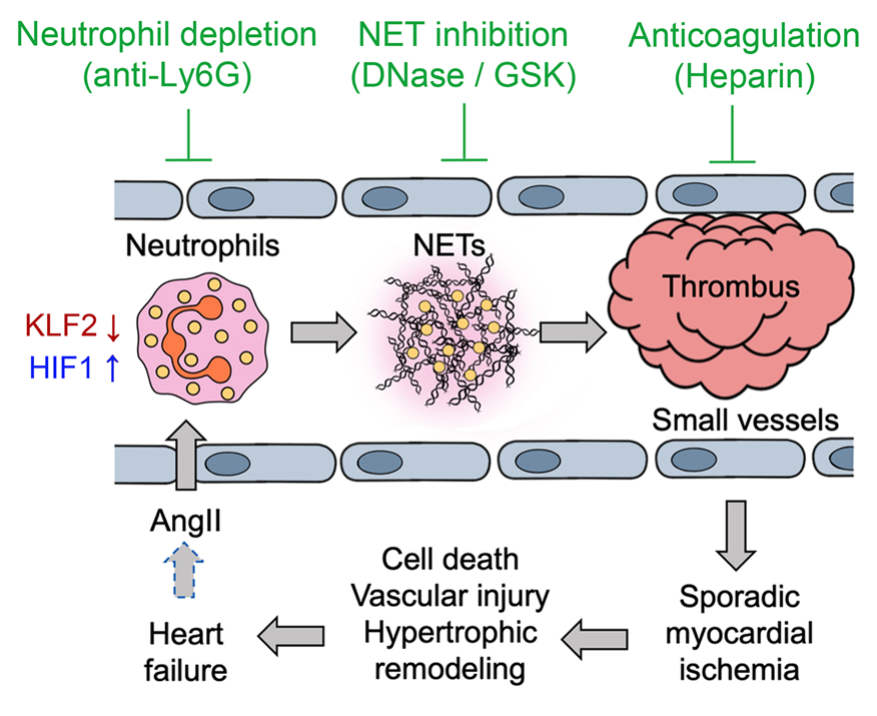
Working model. AngII-induced NETosis results in microthrombosis and sporadic ischemia in the myocardium, promoting cardiac hypertrophy. In HF patients, hyperphysiological AngII levels due to a heightened renin-angiotensin system may propel this vicious cycle (dashed arrow). This model suggests novel therapeutic approaches for HF by targeting neutrophils, NETs, or thrombosis.

The pathogenesis and progression of HF are multifactorial, and accumulating evidence suggests that inflammatory cells and related proinflammatory cytokines play a pathogenic role. In the present study, using chronic AngII infusion as a classic nonischemic HF model, we show a requisite role of neutrophils in cardiac dysfunction (summarized in Figure 12). Our data demonstrate that AngII activates neutrophils to mediate cardiac hypertrophy through a KLF2/NETosis/thrombosis pathway. Activated neutrophils adhere to vascular walls and release NETs, leading to thrombotic occlusion of small vessels and impaired myocardial microcirculation. Chronic microthrombosis may cause capillary rarefaction to further worsen the hypoxic condition in the myocardium. In HF patients, the hyperphysiological levels of AngII due to heightened RAS activity may propel this vicious cycle during HF pathogenesis and progression (Figure 12, dashed arrow). Our model underscores clinical findings showing that small vessel dysfunction is a strong independent predictor of HF deterioration and death, regardless of ischemic or nonischemic HF (51–54). Identifying a neutrophil/KLF2/NETosis/thrombosis pathway for chronic nonischemic cardiac diseases provides pathogenic mechanisms and promising therapeutic targets for HF.

An essential aspect of our work relates to identifying neutrophils as critical regulators of chronic nonischemic heart diseases. Surprisingly, despite being the predominant leukocytes in human blood, little is known about neutrophils in HF. It has been reported that, as the first responders to injury, neutrophils are recruited to the heart during AMI to mediate wound healing (16). Neutrophils are also found in AMI thrombi, suggesting they may participate in thrombosis that causes AMI (55). However, the role of neutrophils in chronic HF and other nonischemic cardiac diseases is poorly understood. Indeed, neutrophils can regulate both acute and chronic inflammation. Several chronic inflammatory and autoimmune diseases are characterized by a sustained influx of neutrophils, such as cystic fibrosis, chronic obstructive pulmonary disease, systemic lupus erythematosus, and rheumatoid arthritis, to name a few (56).

Moreover, metabolic diseases, such as diabetes and obesity that pose risk for HF, are associated with persistent low-grade neutrophil activation and NETosis (55). COVID-19 is also associated with HF, and a high neutrophil/lymphocyte ratio is a predictor of severe outcomes (57). This study found that neutrophils are activated by chronic AngII infusion, likely via neutrophil-autonomous KLF2-dependent signaling, as suggested by in vitro findings (Figure 1, D and E). Meanwhile, cardiac tissue injury triggered by AngII-dependent or AngII-independent mechanisms can also indirectly recruit and activate neutrophils in the heart. Future studies will be needed to delineate the detailed mechanisms by which neutrophils are activated in the course of HF.

Nonetheless, neutrophil activation leads to long-term effects on cardiac hypertrophy in our mouse model, likely through a KLF2/NETosis pathway that is also associated with HF in patients. Our findings are aligned with the clinical observations that the neutrophil/lymphocyte ratio is a prognostic marker for acute and chronic HF hospitalization and mortality. Furthermore, the beneficial effects of ACE inhibitors in HF might be partly due to effects on neutrophils, including antiinflammation and neutropenia (58). Given that neutrophils are the most abundant leukocytes in human blood, identifying neutrophils as crucial regulators of HF is of great importance for basic research and clinical applications.

Recently, studies from our group and others have revealed the distinct roles of monocytes and macrophages in the heart (59). Although we found that KLF2 deficiency in macrophages was not the critical determinant of AngII-induced cardiac hypertrophy in this study, one should not exclude the functions of macrophages in HF. On the one hand, numerous studies using the same AngII model and other cardiac models have shown that macrophages exhibit profibrotic, proinflammatory roles in the heart (60). Our scRNA-Seq data demonstrated the proinflammatory activation of cardiac macrophages, which was exacerbated in the K2KO group (Figure 11A and Supplemental Figure 12). On the other hand, we found that alterations in neutrophils (KLF2 deficiency, neutrophil depletion, DNase I treatment, etc.) did not affect cardiac macrophage numbers. This, however, does not mean that the functions of cardiac macrophages are not affected by neutrophils. It has been reported that neutrophils regulate macrophage functions in response to infection or tissue injury (61, 62). Finally, AngII (and AngII-induced cytokines) can also activate signal transduction in macrophages unrelated to neutrophils (60). For example, we showed that AngII did not induce neutrophil infiltration to KLF2-HIF1α-DKO hearts, but it significantly increased the cardiac macrophage numbers (Figure 9E).

Given that depletion of neutrophils or neutrophil-derived products (NETs, microthrombi) profoundly ameliorated HF, our findings suggest neutrophils may function upstream of macrophages and monocytes in the development of HF. In this study, we also explored the crosstalk between neutrophils and other cells in the myocardium by the scRNA-Seq approach. Major immune cell types identified from the myocardium are macrophages, neutrophils, and lymphocytes, with significant changes in neutrophil numbers between genotypes. Furthermore, the interactome analysis suggests that neutrophils are the key regulator of myocardial inflammation because KLF2-deficient neutrophils, which exhibit enhanced proinflammatory functions, significantly reshape the immune responses from all major cell types in the K2KO myocardium (Figure 11B). Future studies are warranted to study the crosstalk between neutrophils and other immune cells in the setting of HF.

Another important aspect of our work relates to neutrophil control of small vessel thrombosis and microcirculation in a chronic nonischemic disease setting. Our studies found that the effects of neutrophils on cardiac hypertrophy were not mediated by paracrine cytokines but rather through the regulation of hypoxic stress in the myocardium. In AngII-infused mice, particularly those with KLF2-deficient proinflammatory neutrophils, we showed strong evidence of sporadic thrombosis in small vessels throughout the myocardium, associated with capillary rarefaction and cell death. Conversely, treatment with the classic anticoagulant heparin prevented thrombosis, cell death, and subsequently improved cardiac function and myocardial pathologies. Further, these defects were also rescued by administering DNase I or GSK-484, indicating a crucial role of NETs. Consistently, depletion of neutrophils also ameliorated these pathological events. Therefore, we proposed a neutrophil-NET-thrombosis hypothesis to interpret these cardiac responses. Based on this hypothesis, AngII-activated neutrophils are recruited to the heart, forming intravascular NETs that trigger thrombosis. In particular, small vessels prone to occlusion are affected the most, leading to sporadic myocardial ischemia, death of cardiomyocytes and vascular ECs, and subsequent capillary rarefaction. These pathological changes are factors known to cause cardiac hypertrophy and fibrosis (63, 64). Collectively, our findings suggest that nonischemic cardiac diseases, such as cardiac overload secondary to hypertension or neurohormonal stress, may share pathogenic characteristics similar to ischemic cardiac disorders. This notion is consistent with the clinical observations of myocardial capillary rarefaction in HF patients (54), as well as experimental studies demonstrating that inadequate myocardial perfusion due to impaired angiogenesis is a critical determinant of transition from compensatory hypertrophy to HF (65–67).

The neutrophil-NET-thrombosis hypothesis is well supported by our data from this study and literature showing that neutrophils are critically involved in thrombotic processes (36). In mouse studies of arterial thrombosis, neutrophils are the first cells at the site of damage, arriving even earlier than platelets (68). Neutrophils are also found in the venous thrombi from a mouse model of inferior vena cava ligation (31). Mechanistically, the prothrombotic activity of neutrophils has been linked to NETs (35). Nucleosomes, significant components of NETs, can directly activate coagulation factors and platelets. The neutrophil-derived serine proteases, elastase, and cathepsin G deposited onto NETs promote proteolysis of the coagulation suppressor TFPI (tissue factor pathway inhibitor), releasing the brakes of coagulation (37, 69). Tissue factor, the initiator of the extrinsic coagulation cascade, is also found on NETs. Fibrin also connects with NETs, which may be critical for immunothrombosis.

Collectively, these data demonstrate that a KLF2/HIF1α axis is operative in neutrophils in response to chronic sterile inflammation. Furthermore, the fact that neutrophil deficiency of KLF2 or HIF1α was able to skew cardiac responses so profoundly suggests that, for each HF patient, the pathogenesis and progression of HF can be dictated by the condition of their immune system. HF affects more than 26 million people worldwide and has a devastating mortality rate comparable to many malignant cancers (70–72). However, current therapies to reduce neurohormonal stress and improve hemodynamics are only partially effective, underscoring the need for orthogonal strategies based on newly identified molecular and cellular mechanisms. The studies here highlight the previously unidentified essential roles of neutrophils in an experimental model of HF. Further, they suggest that inhibiting neutrophil activation, blocking NET formation or promoting NET clearance, or targeting the downstream microthrombosis, can be exploited for therapeutic gain in the clinical management of HF.

## Methods

Detailed materials and methods can be found in the supplemental material. RNA-Seq data have been deposited in the NCBI’s Gene Expression Omnibus (bulk RNA-Seq data accession no. GSE186468; scRNA-Seq data accession no. GSE185756).

### Study approval.

All animal experimental procedures were approved by the Institutional Animal Care and Use Committee of Case Western Reserve University School of Medicine and were conducted in accordance with the NIH *Guide for the Care and Use of Laboratory Animals* (National Academies Press, 2011). The human study was approved by the Institutional Review Board of Shanghai Minhang Hospital of Integrated Traditional Chinese and Western Medicine (2019005). Written informed consent was obtained from all the participants included in the study.

## Author contributions

XT, RZ, IW, EC, VV, LN, S Lapping, S Liao, AM, DRS, and XL performed animal-based and cell-based experiments and analyzed the data. XT, PW, JL, JF, and TZ carried out human studies and analyzed data. VV, HWJ, and RHA performed bioinformatics. XL conceived the project. TZ, XL, and MKJ supervised the project. XL, MKJ, TZ, and XT wrote the manuscript with input from all authors.

## Supplementary Material

Supplemental data

## Version 1 11/18/2021

In-Press Preview

## Version 2 02/01/2022

Electronic publication
