# Supplementary material for: KLF2 regulates neutrophil activation and thrombosis in cardiac hypertrophy and heart failure progression
Source: J Clin Invest. 2022 Feb 1;132(3):e147191. doi: 10.1172/JCI147191 (PMC8803339; doi:10.1172/JCI147191)
Supplement: Supplemental data [file jci-132-147191-s218.pdf]

Supplemental Information

KLF2 regulates neutrophil activation and thrombosis in cardiac hypertrophy and heart failure progression

Xinmiao Tang[1,2], Peiwei Wang[1,3], Rongli Zhang[2], Ippei Watanabe[2], Eugene Chang[2], Vinesh Vinayachandran[2], Lalitha Nayak[2], Stephanie Lapping[2], Sarah Liao[2], Annmarie Madera[2], David R. Sweet[2], Jiemeng Luo[4], Jinsong Fei[4], Hyun Woo Jeong[5], Ralf H. Adams[5], Teng Zhang[1,3,] *, Xudong Liao[2,] *, Mukesh K. Jain[2,] *

1. Yueyang Hospital, Shanghai University of Traditional Chinese Medicine, Shanghai 200437, China.
2. Case Cardiovascular Research Institute, Case Western Reserve University School of Medicine, Harrington Heart and Vascular Institute, University Hospitals Cleveland Medical Center, Cleveland, Ohio 44106, USA
3. Clinical Research Institute of Integrative Medicine, Shanghai Academy of Traditional Chinese Medicine, Shanghai 200437, China.
4. Minhang Hospital of Integrated Traditional Chinese and Western Medicine, Shanghai 200241, China.
5. Max Planck Institute for Molecular Biomedicine, Department of Tissue Morphogenesis, 48149 Münster, Germany.

* Address correspondence to:
Mukesh K. Jain, MD, Case Western Reserve University, School of Medicine, Cardiovascular Research Institute, Wolstein Research Building, 2103 Cornell Road, WRB 4-522, Cleveland, Ohio 44106, USA. Phone: 1-216-368-3607; Email: mukesh.jain2@case.edu

Xudong Liao, PhD, Case Western Reserve University, School of Medicine, Cardiovascular Research Institute, Wolstein Research Building, 2103 Cornell Road, WRB 4-528A, Cleveland, Ohio 44106, USA. Phone: 1-216-368-3581; Email: xudong.liao@case.edu

Teng Zhang, MD, PhD, Yueyang Hospital & Clinical Research Institute of Integrative Medicine, Shanghai University of Traditional Chinese Medicine, 110 Ganhe Rd, Shanghai 200437, China. Phone: +86-21-55982301; E-mail: zhangteng2089@shutcm.edu.cn

Supplemental methods

Human subjects

Whole blood was collected from a total of 15 heart failure patients diagnosed at the Department of Cardiology, Shanghai Minhang Hospital of Integrated Traditional Chinese and Western Medicine from December 1st, 2019 to March 31st, 2020 and January 1st, 2021 to April 30th, 2021. The diagnosis of heart failure was made based on the criteria of the 2017 ACC/AHA/HFSA Focused Update of the 2013 ACCF/AHA Guideline for the Management of Heart Failure (1). In addition, whole blood from 16 volunteers free of heart failure diagnosis and identifiable infectious and autoimmune diseases was also collected to serve as non-heart failure controls. Peripheral blood leukocytes, neutrophils, and plasma specimens were isolated and kept at -80 °C until indicated analyses were performed. The study was approved by the Institutional Review Board of Shanghai Minhang Hospital of Integrated Traditional Chinese and Western Medicine (2019005). Informed consent was obtained from all the participants included in the current study.

Animals

All mice are on a C57BL/6J background. Myeloid KLF2 deficient mice (Lyz2-Cre:Klf2$^{fl/fl}$, K2KO) were described previously (2). The K2KO line was further crossed to Hif1α-flox mice to generate Lyz2-KLF2-Hif1α-DKO (DKO). The Cx3cr1-K2KO line was developed by crossing Cx3cr1-Cre to KLF2-flox (3). The Lyz2-Cre and Cx3cr1-Cre lines were used as genetic controls, respectively. WT, Hif1α-flox and Cx3cr1-Cre mouse lines were purchased from the Jackson laboratory. The 3xFLAG knock-in KLF2-tag mice were generated using the CRISPR/Cas9 method via collaboration with Cyagen Biosciences. Mice were housed in a temperature- and humidity-controlled specific pathogen-free facility with a 12h-light/dark cycle and ad libitum access to water and chow. Neonatal Sprague Dawley (SD) rats used for cardiac cell isolation were from purchased from Charles River Laboratories International, Inc. Animal studies were approved by the Institutional Animal Care and Use Committee of Case Western Reserve University.

Primary cells isolation and culture

Human and mouse peripheral blood leukocytes and neutrophils were prepared from whole blood and directly subjected to RNA or protein extraction without further in vitro culture. Neonatal rat ventricular myocytes (NRVM) and cardiac fibroblasts were isolated from Day-2 SD rat pups and cultured as described previously (4). Mouse bone marrow-derived macrophages (BMDM) were differentiated in DMEM containing 10% FBS and 25% L-929 conditioned medium (5). Mouse bone marrow neutrophils were isolated by Histopaque-1119/1077 density gradient centrifugation (6). In

brief, bone marrow cells were collected from femurs and tibias, filtered through a 100 µm cell strainer, washed with 10 ml cold HBSS, and collected by centrifugation (300 g). Cells pellets were resuspended in 1 ml HBSS, treated with 20 ml 0.2% NaCl to lysis red blood cells for 20 seconds, and then neutralized with 20 ml 1.6% NaCl. Next, the collected cells were resuspended in 1-3 ml HBSS and overlay on 3 ml Histopaque 1119 (Sigma-Aldrich, 11191) and 3 ml Histopaque 1077 (Sigma-Aldrich, 10771), followed by 30 min centrifugation at 900 g at 25 °C with lowest settings of acceleration and brake. Neutrophils were collected from the interface of the Histopaque-1119 and 1077. Finally, samples were washed with 10 ml cold HBSS, counted, and the pellets were resuspended in either HBSS or medium at the desired concentration.

Mouse models for neutrophil transfusion and depletion

Five million freshly purified mouse bone marrow neutrophils were re-suspended in 100 µl medium and transfused into the recipient mouse via intravenous injection (*i.v.*). Neutrophil depletion was achieved by injection (i.v.) of anti-Ly6G antibody (clone 1A8, Bio X Cell, BP0075-1) at 4 mg/kg every other day, starting from 1 day before surgery (7). Normal rat IgG (clone 2A3, Bio X Cell, BP0089) was used as control.

Mouse Angiotensin II infusion cardiac hypertrophy model

Angiotensin II (Sigma-Aldrich, A9525) was administered to male mice for 1-4 weeks at 1.0 µg/kg/min or 2.0 µg/kg/min as indicated using subcutaneous osmotic pumps (Alzet). In brief, mice were anesthetized by intraperitoneal (*i.p.*) injection of a ketamine xylazine cocktail (prepared by University's Animal Resource Center) and a small incision (5-10 mm) was made on the back to dissect a skin area for the subcutaneous implantation of mini-pumps filled with AngII or PBS. Buprenorphine was administrated daily for the first three days post-surgery. For echocardiography, mice were anesthetized by inhalation of 0.1-0.5% isoflurane vaporized in 100% oxygen keeping a steady heart rate above 500 bpm, and LV recording was obtained at parasternal short axis view in B- and M-mode on a Vevo-770 or 3100 System (Fujifilm) (8).

Assessment of blood pressure by invasive hemodynamics

Invasive hemodynamics was performed as previously described (9). Mice were anesthetized with 0.25 mg/g (*i.p.* injection) 2,2,2-Tribromoethanol (prepared by Animal Resource Center) and respiratory support was supplied with a rodent ventilator (MiniVent 845; Harvard Apparatus). A Millar SPR-1000 catheter was inserted into the right common carotid artery to measure blood pressure. Pressure data were obtained with an MPVS-300 system (Millar Instruments) coupled to a PowerLab data acquisition system and calculated using LabChart (ADInstruments).

Assessment of myocardial microcirculation by contrast-ECHO

Mice were anesthetized by 2,2,2-Tribromoethanol (*i.p.* injection), which dose was carefully titrated to maintain the heart rate at 400-500 bpm. Using an *i.v.* catheter, contrast agent (Vevo MicroMarker Non-Targeted Contrast Agent, VS-11913, 1: 10 dilution with saline) was infused at 20 uL/min. Transthoracic ECHO was performed with a Vevo MX201 transducer on a Vevo 3100 system (Fujifilm), using the Non-Linear Contrast (NLS) function in long-axis B mode. To record still images for data analysis, ECHO was gated on ECG. About 2 min into the infusion, ECHO detected a stable level of the contrast signal. At 3-min post-infusion, a transient high-energy ultrasound beam was applied by the "Burst" function. ECHO was continuously recorded starting from 2.5-min of infusion (before Burst) until the stable level of contrast signal was re-established (Recovery). ECHO images were analyzed in VevoLab software to calculate time-stamped contrast signals (Density-Time data) in the defined region of interest (ROI), the LV anterior wall in this study. Curve fitting was performed in Prism 8 software using the one-phase decay exponential fitting method, and time constant Tau was calculated to estimate the recovery rate.

NETs inhibition in vivo by DNase I or GSK-484 treatment

Deoxyribonuclease I (Worthington Biochemical Corporation, LS002007) was diluted in PBS and administered at 4 mg/kg (in 100 µL PBS) by *i.p.* injection after AngII subcutaneous pump implantation, followed by daily dose. PBS (100 µL) was injected as the vehicle control.

GSK-484 (hydrochloride, Cayman Chemical, 17488), a reversible PAD4 inhibitor, was dissolved in 100% ethanol at 25 mg/ml as stock solution and diluted 1:50 with PBS before injection. For GSK-484 in vivo treatment, mice were treated with GSK-484 (4 mg/kg) or vehicle (2% ethanol in PBS) through *i.p.* injection after AngII pump subcutaneous implantation, followed by daily dose.

Flow cytometry analysis and sorting

The mouse heart was perfused, excised, minced, digested with type I collagenase, mechanically disrupted, and filtered through a 70 µm cell strainer to get a single cell suspension (7). Cells were collected by centrifugation (700 g), subjected to staining with live-dead dye (Invitrogen, L34968) and fluorescent surface antibodies, and analyzed by flow cytometry. Dead cells were excluded by live-dead stain positivity. Live cells were also sorted for transcriptomic studies.

Fluorescent antibodies used in this study: CD45-PerCP (BD Biosciences, 561047), CD11b-AF488 (BD Biosciences, 557672), Ly6G-APC-Cy7 (BD Biosciences, 560600), F4/80-BV421 (BioLegend, 123137), CD45-FITC (BioLegend, 103108), CD31-BV421 (BioLegend, 123137).

Histology

Cardiac tissue samples were fixed in 10% neutralized formalin and embedded with paraffin following standard protocols. Fibrosis was visualized using Picrosirius Red Stain Kit (Polysciences, 24901.) (8). TUNEL staining was performed with the ApopTag Fluorescent In Situ Apoptosis Detection Kit (Millipore, S7110) (4). Capillary staining was performed in cryopreserved heart sections using an anti-CD31 primary antibody (1:50, BD Biosciences, 553370) and Alexa Fluor 594-conjugated goat-anti-rat secondary antibody (Invitrogen, A11007). Cardiomyocyte cross-sectional area was determined by staining with fluorescence-conjugated Wheat Germ Agglutinin (WGA-AF594/AF488) (ThermoFisher Scientific, W11262, W11261). NETs staining was performed with cryopreserved heart sections using rabbit polyclonal anti-Histone H3 (citrulline R2 + R8 + R17) primary antibody (1:1000, Abcam, Ab5103), and Alexa Fluor 488-conjugated goat-anti-rabbit secondary antibody (Invitrogen, A11008). Microthrombi were stained with primary antibodies for P-selectin (2 μg/ml, BioLegend, 148301, mouse antibody) and vWF (1:100, Invitrogen, PA5-16634, rabbit antibody), and followed by staining with fluorescent anti-mouse and anti-rabbit secondary antibodies (Invitrogen, A11032, A11008). Hif1α protein was stained with anti-HIF1α primary antibody (1:100, Cell Signaling, D2U3T) and Alexa Fluor 488-conjugated anti-rabbit secondary antibody. Microscopic images were analyzed using NIH ImageJ software.

Quantification of NETosis-related biomarkers in the plasma

A fluorometric assay for double-stranded DNA analysis was performed to measure the plasma level of cell-free DNA (cfDNA) using the Quant-iT PicoGreen dsDNA Assay Kit (ThermoFisher Scientific, P11496) following the manufacturer's instructions. In addition, the plasma level of histone-associated DNA fragments was analyzed using the Cell Death Detection ELISA Kit (Roche Diagnostics, 11544675001) according to the manufacture's protocols. Absorption was detected at 450 nm (OD450).

RNA extraction and qPCR

Total RNA was purified using the Qiagen RNeasy kits (Qiagen, 74004, 74104). Tissue samples were homogenized in the RLT lysis reagent with a Qiagen TissueLyser II homogenizer (Qiagen), and cell samples were directly dissolved in the RLT lysis reagent. RNA was reverse transcribed to complementary DNA using the iScript Reverse Transcription Kit (Bio-Rad, 170-8841). Quantitative real-time PCR (qPCR) was performed with either the TaqMan method (Roche Universal ProbeLibrary System) or the SYBR green method. Relative expression was calculated using the ΔΔCt method with normalization to Gapdh.

Bulk RNA-seq studies

Neutrophils were FACS sorted from mouse myocardium as the CD45+CD11b+Ly6G+ cell population. Dead cells were excluded by the live-dead dye. RNA-Seq libraries were prepared from ~5000 sorted neutrophils using the Illumina TruSeq Low-input Total RNA Sample Preparation kit according to the manufacturer's protocol. Seventy-five bp paired-end sequencing was performed on pooled libraries using an Illumina HiSeq 2500. Data analysis was performed following an in-house pipeline established at CWRU Genomics Core as previously described (7).

Single-cell RNA-seq studies

Single-cell library construction and sequencing

CD45+ cells and CD31+ cells were sorted from mouse myocardium by flow cytometry. Dead cells were excluded by the live-dead dye. Totally 75,000 CD45+ cells and 75,000 CD31+ cells (1:1) were input to the 10xGenomics Chromium controller for single-cell partitioning and Gel Bead-in-Emulsion (GEM) generation. GEMs are reaction vessels in which cDNA from each single cell is generated and uniquely barcoded. cDNA libraries were checked for quality using an Agilent 2100 Bioanalyzer. Adapters were ligated, and barcoding was accomplished for each library, followed by an additional assessment of average fragment size using the bioanalyzer. Samples were pooled and sequenced on a Novaseq 6000 S4 flow cell using the sequencing parameters outlined in the 10x protocol (version CG000204).

Single-cell RNA-seq analysis

FASTQ format of raw sequencing data were processed with UMI-tools (version 1.0.0) to generate a whitelist for cell barcodes and UMIs and to extract valid sequencing reads, aligned to the mouse reference genome (mm10) with STAR (version 2.7.3a), and quantified with Subread featureCounts (version 1.6.4). Data normalization, cell clustering, and visualization were performed using Seurat (version 3.1.5). For initial quality control of the extracted gene-cell matrices, we filtered cells with parameters low.threshold = 500, high.threshold =6,000 for number of genes per cell (nFeature_RNA), high.threshold = 25% for percentage of mitochondrial genes (percent.mito) and genes with parameter min.cell =3. Filtered matrices were normalized by LogNormalize method with scale factor=10,000. Variable genes were found with parameters of selection.method = vst and nfeatures = 2,000, trimmed for the genes related to cell cycle (GO:0007049) and then used for data integration (IntegrateData with FindIntegrationAnchors function), data scaling (ScaleData), and principal component analysis (RunPCA). Statistically significant principal components were determined by the JackStraw method, and the first 9

principle components were used for non-linear dimensional reduction (UMAP) and clustering analysis (FindNeighbors) with resolution=0.1.

GO terms enrichment analysis

Differentially expressed genes (DEGs) for each cluster between Cre vs. K2KO samples were identified using the FindAllMarkers function of the Seurat package with options of only.pos=TRUE, min.pct = 0.25 and logfc.threshold = 0. Enriched gene ontologies of molecular function, biological function, and cellular component were identified using enrichGO function of clusterProfiler R package with a q value cutoff of 0.1 and then simplified by removing redundancy of enrich terms with simplify function with cutoff = 0.7, by = p.adjust and measure = Wang options. Top GO terms for each sample were selected by adjusted p values.

Cell-cell interactome analysis

An R package iTALK (doi: https://doi.org/10.1101/507871) was used for the ligand-receptor interactome analysis. Differentially expressed genes for each cluster between Cre vs. K2KO samples were identified using the non-parameteric Wilcoxon rank sum test and used for the detection of ligand-receptor pairs using FindLR function. The results were plotted by LRPlot function with datatype = DEG.

Statistics

Results are presented as mean ± SEM. A two-tailed Student's t test was used to compare the differences between two groups. One-way ANOVA was used for simple multiple comparisons. Two-way ANOVA was used for studies with two independent variables. Post-hoc test with Tukey correction was applied to multiple comparisons. Statistical significance was defined as $P < 0.05$. Statistics was performed using the Graphpad Prism 8 software.

Reference:

1. Yancy CW, Jessup M, Bozkurt B, Butler J, Casey DE, Jr., Colvin MM, et al. 2017 ACC/AHA/HFSA Focused Update of the 2013 ACCF/AHA Guideline for the Management of Heart Failure: A Report of the American College of Cardiology/American Heart Association Task Force on Clinical Practice Guidelines and the Heart Failure Society of America. *Circulation.* 2017;136(6):e137-e61.
2. Mahabeleshwar GH, Kawanami D, Sharma N, Takami Y, Zhou G, Shi H, et al. The myeloid transcription factor KLF2 regulates the host response to polymicrobial infection and endotoxic shock. *Immunity.* 2011;34(5):715-28.
3. Hulsmans M, Clauss S, Xiao L, Aguirre AD, King KR, Hanley A, et al. Macrophages Facilitate Electrical Conduction in the Heart. *Cell.* 2017;169(3):510-22 e20.
4. Liao X, Haldar SM, Lu Y, Jeyaraj D, Paruchuri K, Nahori M, et al. Kruppel-like factor 4 regulates pressure-induced cardiac hypertrophy. *J Mol Cell Cardiol.* 2010;49(2):334-8.
5. Liao X, Sharma N, Kapadia F, Zhou G, Lu Y, Hong H, et al. Kruppel-like factor 4 regulates macrophage polarization. *J Clin Invest.* 2011;121(7):2736-49.
6. Shen Y, Hong H, Sangwung P, Lapping S, Nayak L, Zhang L, et al. Kruppel-like factor 4 regulates neutrophil activation. *Blood Adv.* 2017;1(11):662-8.
7. Liao X, Shen Y, Zhang R, Sugi K, Vasudevan NT, Alaiti MA, et al. Distinct roles of resident and nonresident macrophages in nonischemic cardiomyopathy. *Proc Natl Acad Sci U S A.* 2018;115(20):E4661-E9.
8. Liao X, Zhang R, Lu Y, Prosdocimo DA, Sangwung P, Zhang L, et al. Kruppel-like factor 4 is critical for transcriptional control of cardiac mitochondrial homeostasis. *J Clin Invest.* 2015;125(9):3461-76.
9. Zhang R, Hess DT, Qian Z, Hausladen A, Fonseca F, Chaube R, et al. Hemoglobin betaCys93 is essential for cardiovascular function and integrated response to hypoxia. *Proc Natl Acad Sci U S A.* 2015;112(20):6425-30.

Supplemental figures and tables

Supplemental Figure 1. AngII induces pro-inflammatory activation of neutrophils and macrophages.
(**A**) Protein levels of cytokines in neutrophil-conditioned medium, assessed by ELISA (n=5-6). (**B**) AngII induced changes in mRNA expression of KLF2 and inflammatory cytokines (n=5-6). P values were from the 2-tailed unpaired Student t test. One way-ANOVA with Tukey correction was applied for (B).

Supplemental Figure 2. Effects of high dose AngII infusion.
(**A**) FACS analysis of cardiac myeloid cells. From myocardial single-cell suspension, macrophages were gated as the CD45+CD11b+F4/80+Ly6G- population (P1); neutrophils were gated as the CD45+CD11b+Ly6G+ population (P2). (**B**) Data summary of FACS studies (n=5-7). (**C**) Kaplan Meier survival curves with regular dose (1.0 μg/kg/min) and high dose (2.0 μg/kg/min) AngII infusion. n=5-20. P values were from post-hoc test of Two-way ANOVA with Tukey correction for (B).

Supplemental Figure 3. KLF2 deficiency in macrophages and monocytes does not enhance AngII-induced cardiac hypertrophy.
Cx3cr1-Cre and Cx3cr1-K2KO mice were subjected to a 4-week AngII infusion (1.0 μg/kg/min). (**A**) LV function (LVEF) was assessed by echocardiography. (**B**) Heart weight was measured at the end of the study to evaluate cardiac hypertrophy. HW: heart weight (mg). BW: body weight (g). P values were from post-hoc test of Two-way ANOVA with Tukey correction. NS indicates not significant. n=4-5 in each group.

Supplemental Figure 4. AngII regulates NETosis in vivo and in vitro.
(**A**) NETs formation in the myocardium detected by H3cit immunofluorescence (n=5). DAPI staining indicated nuclei. These are single-channel images for Figure 4A and the merged images were shown in Figure 4A. (**B**) AngII triggered NETs formation in isolated mouse bone marrow neutrophils in vitro (n=6). (**C**) Effects of DNase I-mediated NETs clearance on myocardial infiltration of neutrophils and macrophages (n=5). Treatment: 1 week. P values were from post-hoc test of Two-way ANOVA with Tukey correction for (A, B) and 2-tailed unpaired Student t test for (C).

Supplemental Figure 5. Potential crosstalk between neutrophils and cardiac cells.

(**A**) NRVM treated with 100 nmol/L AngII for 24h, with or without a conditional medium (CM) from neutrophils treated with 100 nmol/L AngII for 0.5, 2, and 4h. n=4-5 in each group. (**B)** CM from neutrophils treated for 0.5h with 10, 100, and 1000 nmol/L AngII. NRVM treated with the same concentrations of AngII for 24h. n=5-6 in each group. (**C**) Expression of collagen genes in cardiac fibroblast with and without treatment of neutrophil CM. n=6 in each group. P values were from post-hoc test of One-way ANOVA with Tukey correction for (A) and Two-way ANOVA with Tukey correction for (B). NS indicates not significant.

Supplemental Figure 6. Immunofluorescent staining with heart sections.

(**A**) Intravascular localization of neutrophils (Ly6G/CD31) and NETs (H3Cit/CD31) in AngII-infused K2KO hearts. (**B**) Intravascular NETs (H3Cit/CD31) and microthrombi (P-selectin/vWF) in AngII-infused K2KO hearts. These are single-channel images for Figure 5A and the merged images were shown in Figure 5A. (**C**) Microthrombi in mouse hearts. These are single-channel images for Figure 5B and the merged images were shown in Figure 5B. Scale bar: 25 µm. AngII infusion: 1-week at 1.0 µg/kg/min. Ly6G: neutrophil marker. CD31: vascular endothelial marker. H3Cit: a marker for NETs. DAPI: a marker for nuclei. Representative images.

Supplemental Figure 7. Depleting neutrophils or clearance of NETs prevents AngII-induced thrombosis, cell death, and capillary rarefaction in K2KO hearts.

(**A**) K2KO mice with anti-Ly6G antibody treatment for neutrophil depletion (n=5). (**B**) K2KO mice with DNase I treatment for NETs clearance (n=5-6). TUNEL data were included in Figure 4D. CD31 staining was from 4-week AngII infusion studies. Others were from 1-week AngII infusion studies. Scale bar: 25 µm. P values were from the 2-tailed unpaired Student t test.

Supplemental Figure 8. Neutrophil depletion attenuates AngII-induced cardiac hypertrophy in WT mice.

WT mice receiving high dose AngII infusion (2.0 µg/kg/min) were injected (*i.v.*) with anti-Ly6G antibody (clone 1A8) to induce neutrophil depletion. Normal IgG was used as a control. n=4-6 in each group. (**A**) Cardiac function assessed by at 4-week post-AngII infusion. (**B**) WGA staining for cardiomyocyte size assessment. (**C**) Expression of marker genes for hypertrophy (*Nppa*) and inflammation in the hearts. (**D**) FACS analysis for myeloid cells in the hearts after 1-week AngII infusion. IgG: normal IgG control. Ly6G: neutrophil depletion by anti-Ly6G. P values were from 2-tailed unpaired Student t test for (A-C) and One-way ANOVA with Tukey correction for (D). NS indicates not significant.

Supplemental Figure 9. Complete blood count after neutrophil transfusion.

Complete blood count was performed with blood harvested from the submandibular vein after 2, 6, and 24 hours of neutrophil infusion. (**A**) Total white cells (n=7). (**B**) Neutrophils (n=7). (**C**) Lymphocytes (n=7). (**D**) Platelets (n=3-7). P values from One-way ANOVA with Tukey correction.

Supplemental Figure 10. RNA-seq study with cardiac neutrophils.

(**A**) Volcano plot showing 1740 differentially expressed genes (DEGs) between Cre and K2KO groups. The dashed line indicates p=0.05. (**B**) GSEA Hallmark pathway enrichment analysis of DEGs. Selected pathways are listed in Figure 9A. Cells sorted from four mice were included as biological replicates for each group (n=4). AngII infusion: 1 week.

Supplemental Figure 11. Single-cell RNA-seq study with cardiac cells.

(**A**) Dotplot for Top 4 marker genes in each cluster. (**B**) Volcano plots showed DEGs in 4 major cell types identified from scRNA-seq. GO analysis was performed using these DEGs in Figure 11. Cells from three mice were pooled before flow sorting in each group (n=3). AngII infusion: 1 week.

Supplemental Figure 12. Neutrophils orchestrate myocardial inflammation and adaptation to AngII stress.

Gene ontology (GO) analyses with Cre DEGs from four major cell types: neutrophils, macrophages, endothelial cells, and fibroblasts, showing Top 10 Biological Process (BP) GO terms according to adjusted P values (p.adjust).

Supplemental Table 1. Patients' information

Supplemental Table 2. Mouse echocardiogram parameters

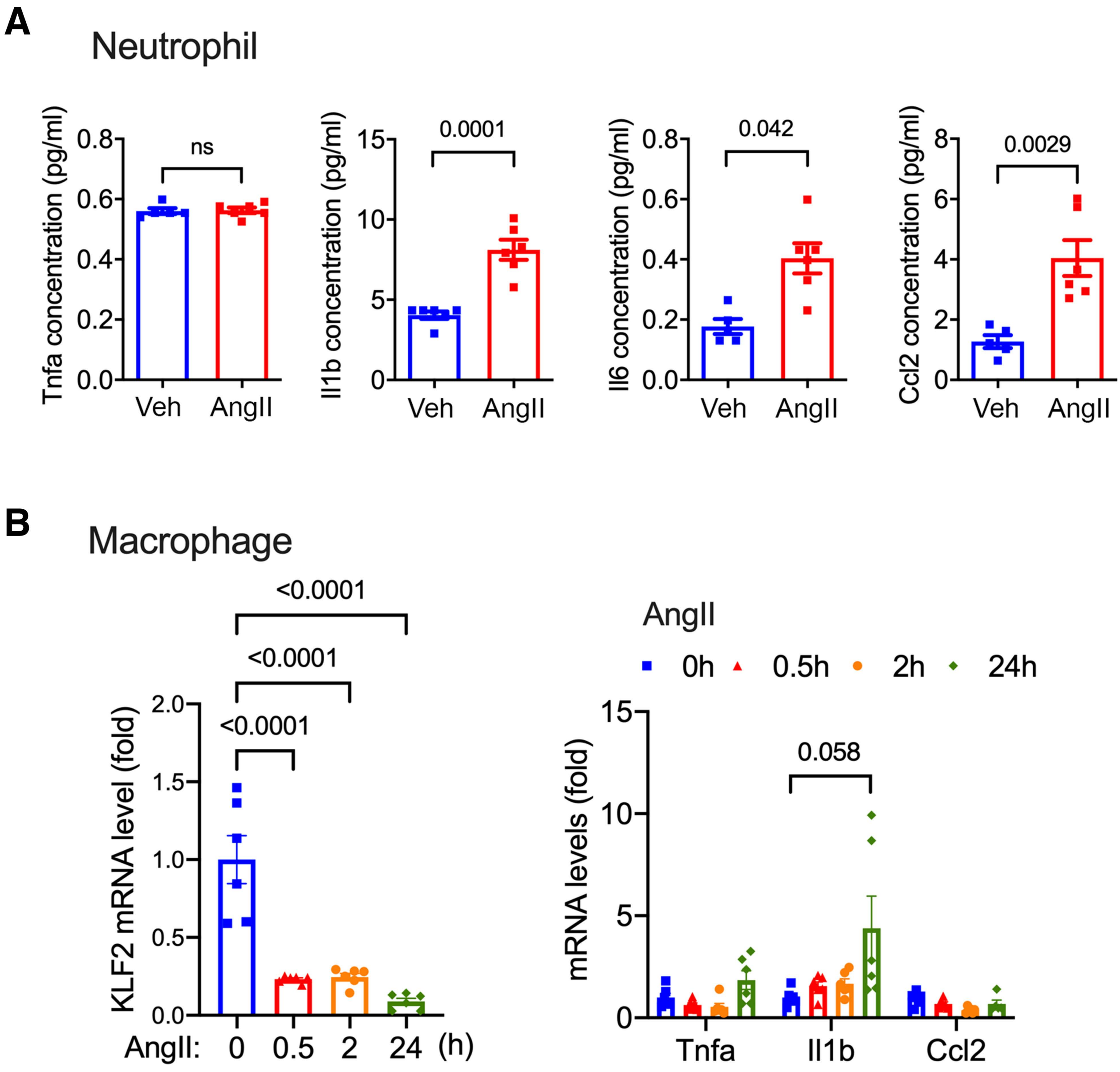

Supplemental Figure 1. AngII induces pro-inflammatory activation of neutrophils and macrophages.
(**A**) Protein levels of cytokines in neutrophil-conditioned medium, assessed by ELISA (n=5-6).
(**B**) AngII induced changes in mRNA expression of KLF2 and inflammatory cytokines (n=5-6).
P values were from the 2-tailed unpaired Student t test. One way-ANOVA with Tukey correction was applied for (B).

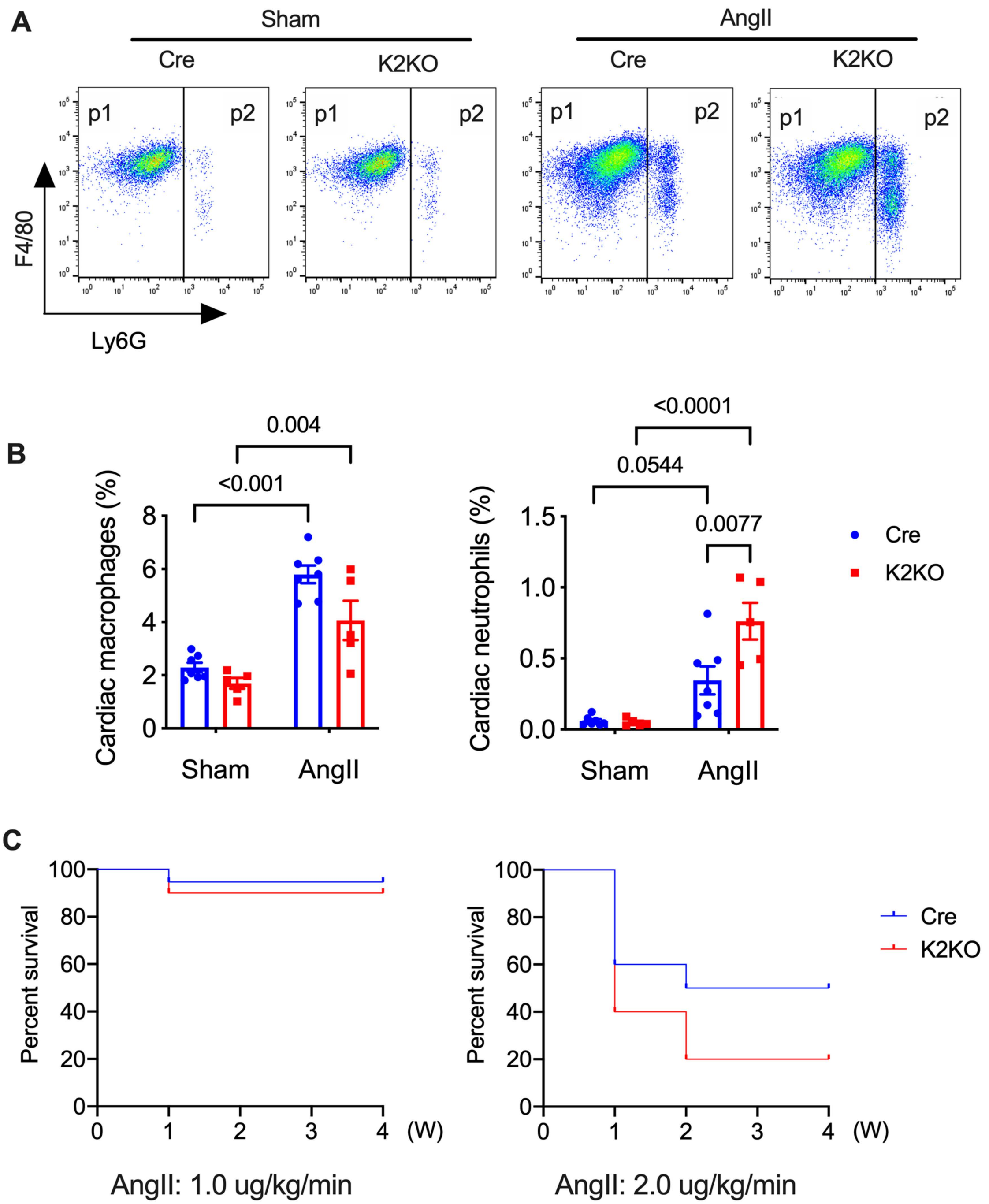

Supplemental Figure 2. Effects of high dose AngII infusion.
(**A**) FACS analysis of cardiac myeloid cells. From myocardial single-cell suspension, macrophages were gated as the CD45+CD11b+F4/80+Ly6G- population (P1); neutrophils were gated as the CD45+CD11b+Ly6G+ population (P2). (**B**) Data summary of FACS studies (n=5-7). (**C**) Kaplan Meier survival curves with regular dose (1.0 μg/kg/min) and high dose (2.0 μg/kg/min) AngII infusion. n=5-20. P values were from post-hoc test of Two-way ANOVA with Tukey correction for (B).

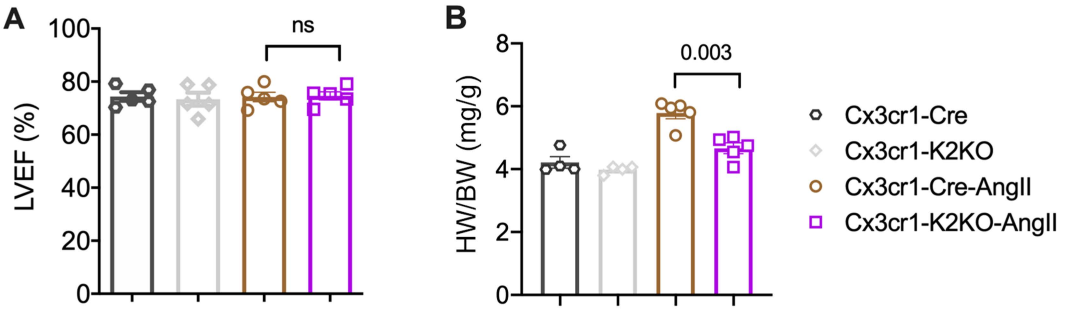

Supplemental Figure 3. KLF2 deficiency in macrophages and monocytes does not enhance AngII-induced cardiac hypertrophy.
Cx3cr1-Cre and Cx3cr1-K2KO mice were subjected to a 4-week Ang**II** infusion (1.0 µg/kg/min). (**A**) LV function (LVEF) was assessed by echocardiography. (**B**) Heart weight was measured at the end of the study to evaluate cardiac hypertrophy. HW: heart weight (mg). BW: body weight (g). P values were from post-hoc test of Two-way ANOVA with Tukey correction. NS indicates not significant. n=4-5 in each group.

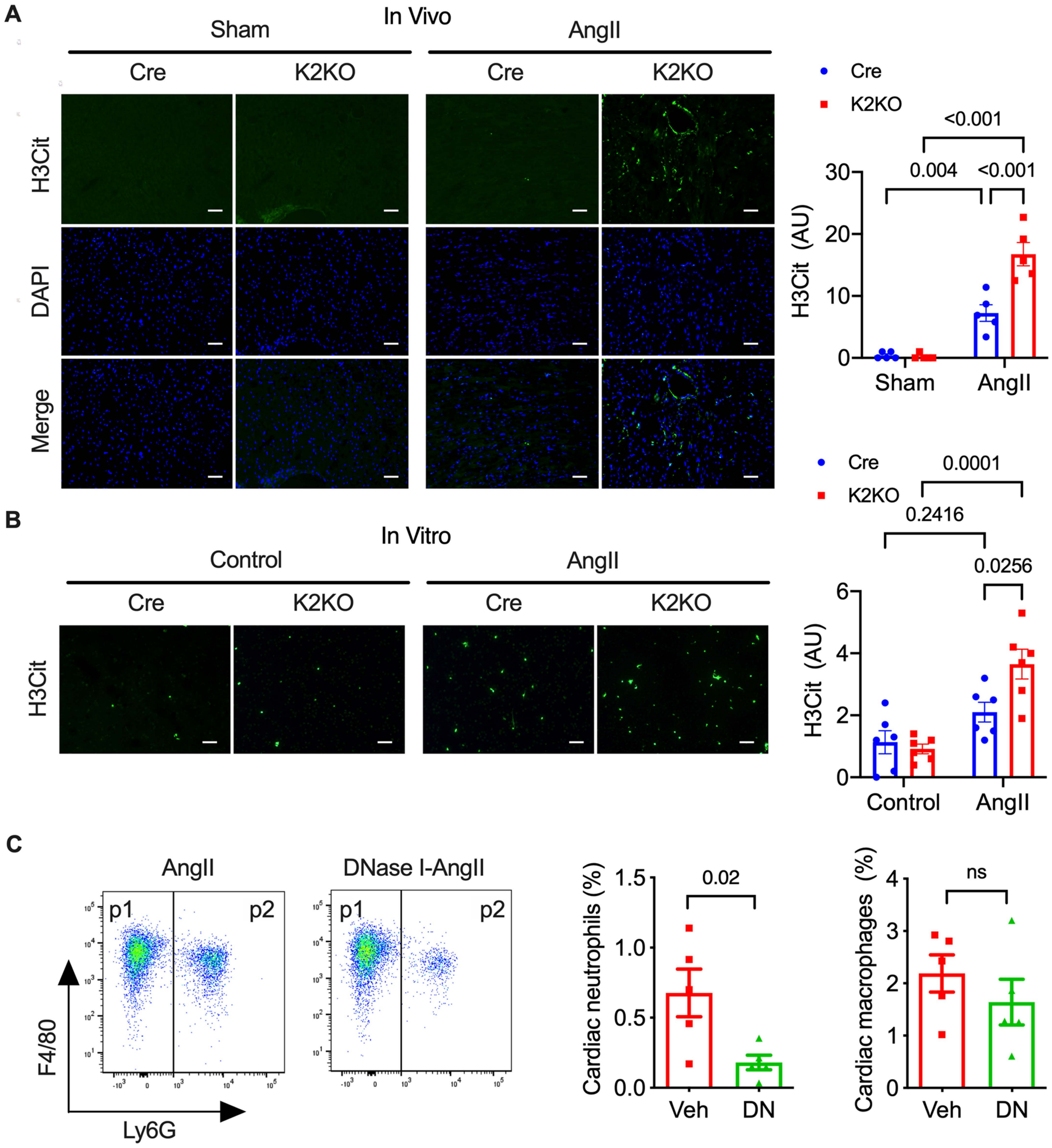

Supplemental Figure 4. AngII regulates NETosis in vivo and in vitro.
(**A**) NETs formation in the myocardium detected by H3cit immunofluorescence (n=5). DAPI staining indicated nuclei. These are single-channel images for Figure 4A and the merged images were shown in Figure 4A. (**B**) AngII triggered NETs formation in isolated mouse bone marrow neutrophils in vitro (n=6). (**C**) Effects of DNase I-mediated NETs clearance on myocardial infiltration of neutrophils and macrophages (n=5). Treatment: 1 week. P values were from post-hoc test of Two-way ANOVA with Tukey correction for (A, B) and 2-tailed unpaired Student t test for (C).

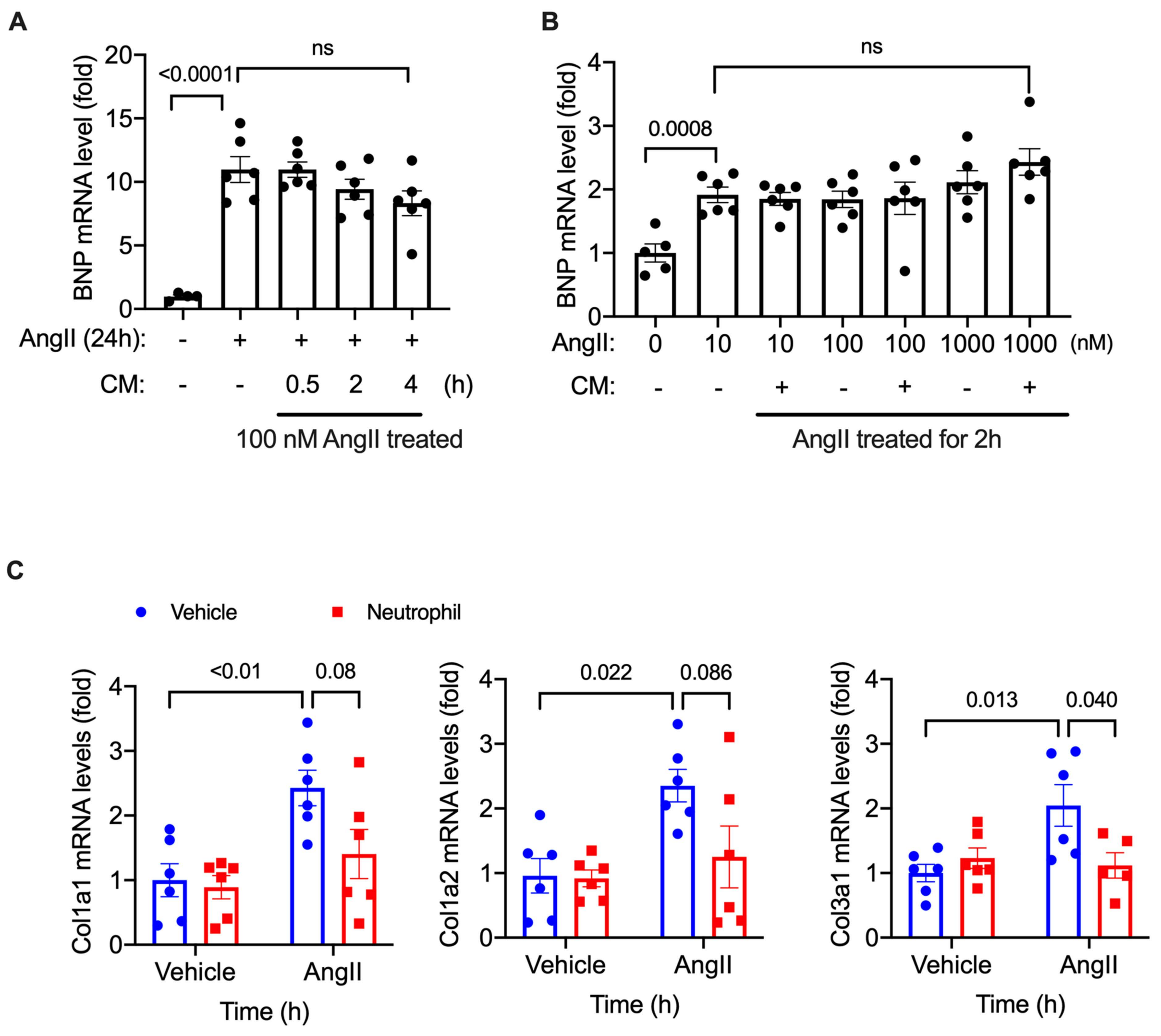

Supplemental Figure 5. Potential crosstalk between neutrophils and cardiac cells.
(**A**) NRVM treated with 100 nmol/L AngII for 24h, with or without a conditional medium (CM) from neutrophils treated with 100 nmol/L AngII for 0.5, 2, and 4h. n=4-5 in each group. (**B**) CM from neutrophils treated for 0.5h with 10, 100, and 1000 nmol/L AngII. NRVM treated with the same concentrations of AngII for 24h. n=5-6 in each group. (**C**) Expression of collagen genes in cardiac fibroblast with and without treatment of neutrophil CM. n=6 in each group. P values were from post-hoc test of One-way ANOVA with Tukey correction for (A) and Two-way ANOVA with Tukey correction for (B). NS indicates not significant.

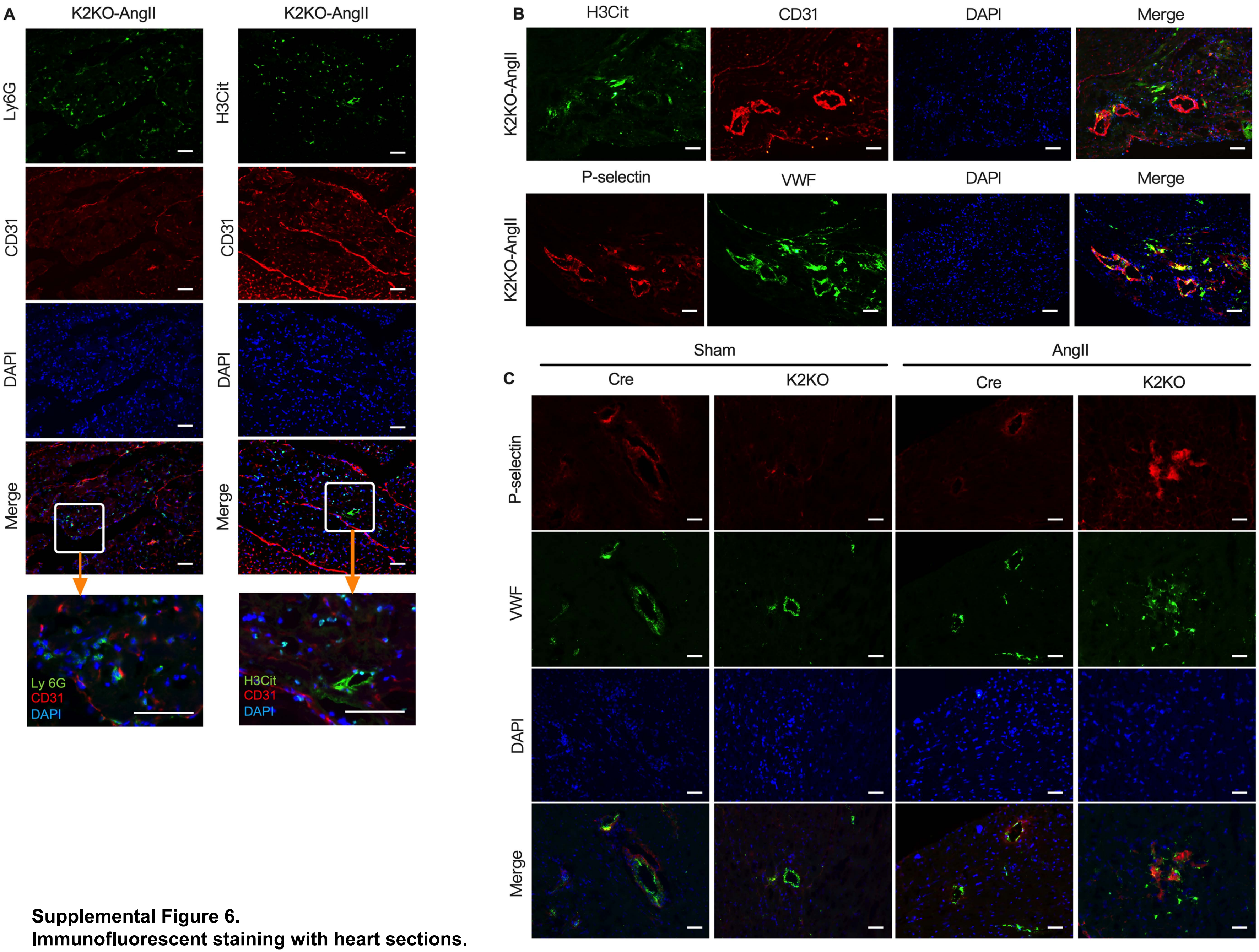

Supplemental Figure 6.
Immunofluorescent staining with heart sections.
(**A**) Intravascular localization of neutrophils (Ly6G/CD31) and NETs (H3Cit/CD31) in AngII-infused K2KO hearts. (**B**) Intravascular NETs (H3Cit/CD31) and microthrombi (P-selectin/vWF) in AngII-infused K2KO hearts. These are single-channel images for Figure 5A and the merged images were shown in Figure 5A. (**C**) Microthrombi in mouse hearts. These are single-channel images for Figure 5B and the merged images were shown in Figure 5B. Scale bar: 25 µm. AngII infusion: 1-week at 1.0 µg/kg/min. Ly6G: neutrophil marker. CD31: vascular endothelial marker. H3Cit: a marker for NETs. DAPI: a marker for nuclei. Representative images.

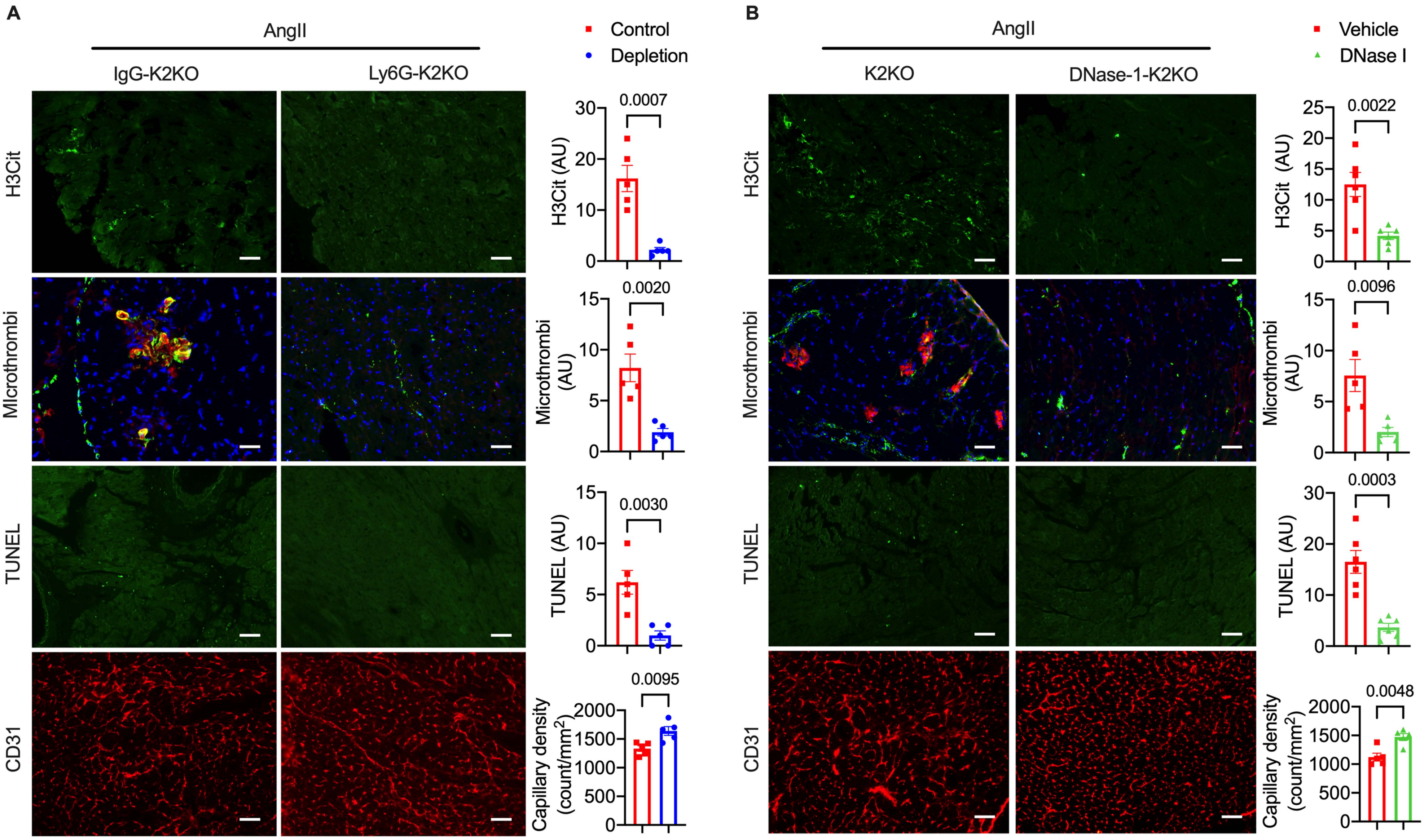

Supplemental Figure 7. Depleting neutrophils or clearance of NETs prevents AngII-induced thrombosis, cell death, and capillary rarefication in K2KO hearts.

(**A**) K2KO mice with anti-Ly6G antibody treatment for neutrophil depletion (n=5). (**B**) K2KO mice with DNase I treatment for NETs clearance (n=5-6). TUNEL data were included in Figure 4D. CD31 staining was from 4-week AngII infusion studies. Others were from 1-week AngII infusion studies. Scale bar: 25 μm. P values were from the 2-tailed unpaired Student t test.

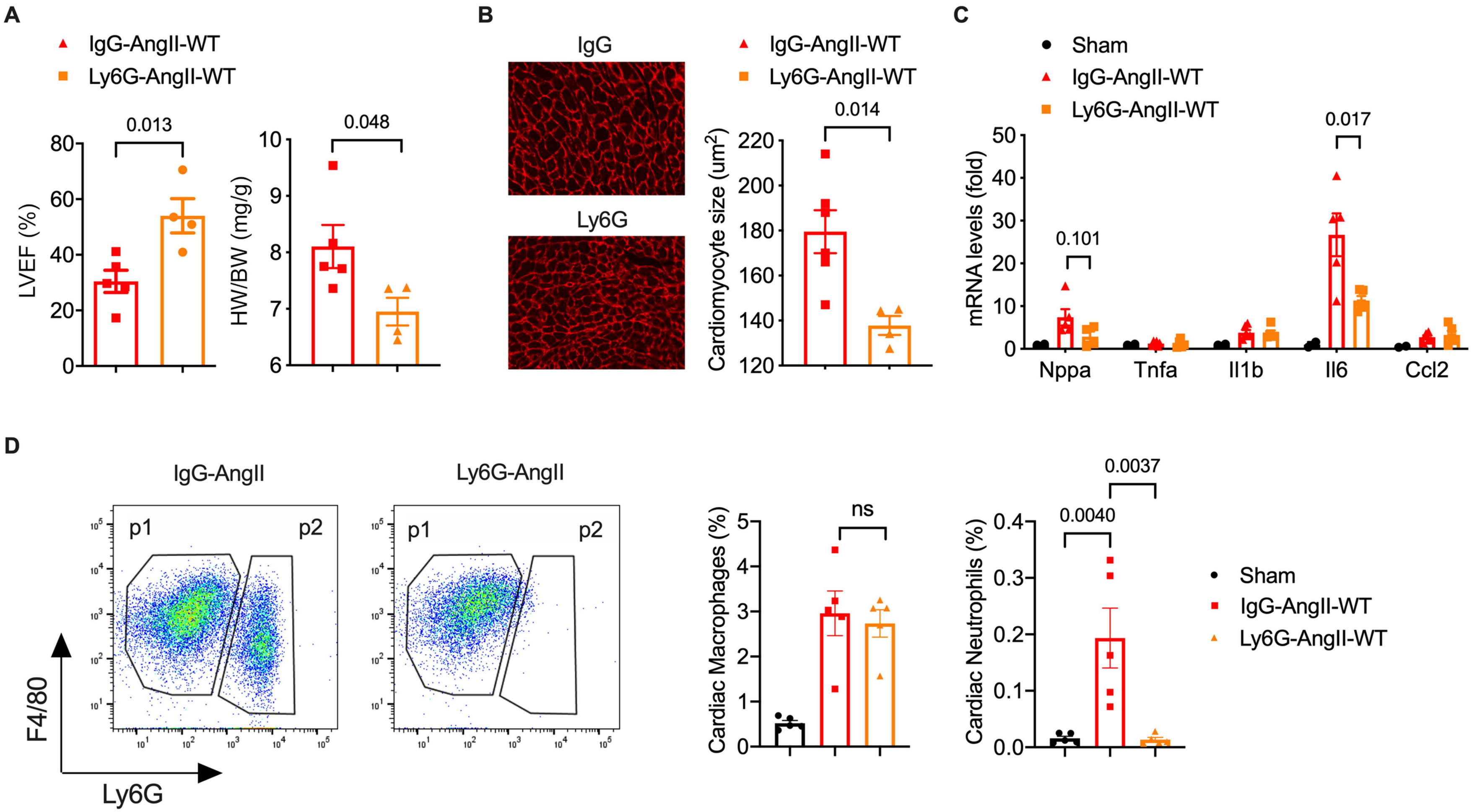

Supplemental Figure 8. Neutrophil depletion attenuates AngII-induced cardiac hypertrophy in WT mice.
WT mice receiving high dose AngII infusion (2.0 μg/kg/min) were injected (i.v.) with anti-Ly6G antibody (clone 1A8) to induce neutrophil depletion. Normal IgG was used as a control. n=4-6 in each group. (**A**) Cardiac function assessed by at 4-week post-AngII infusion. (**B**) WGA staining for cardiomyocyte size assessment. (**C**) Expression of marker genes for hypertrophy (Nppa) and inflammation in the hearts. (**D**) FACS analysis for myeloid cells in the hearts after 1-week AngII infusion. IgG: normal IgG control. Ly6G: neutrophil depletion by anti-Ly6G. P values were from 2-tailed unpaired Student t test for (A-C) and One-way ANOVA with Tukey correction for (D). NS indicates not significant.

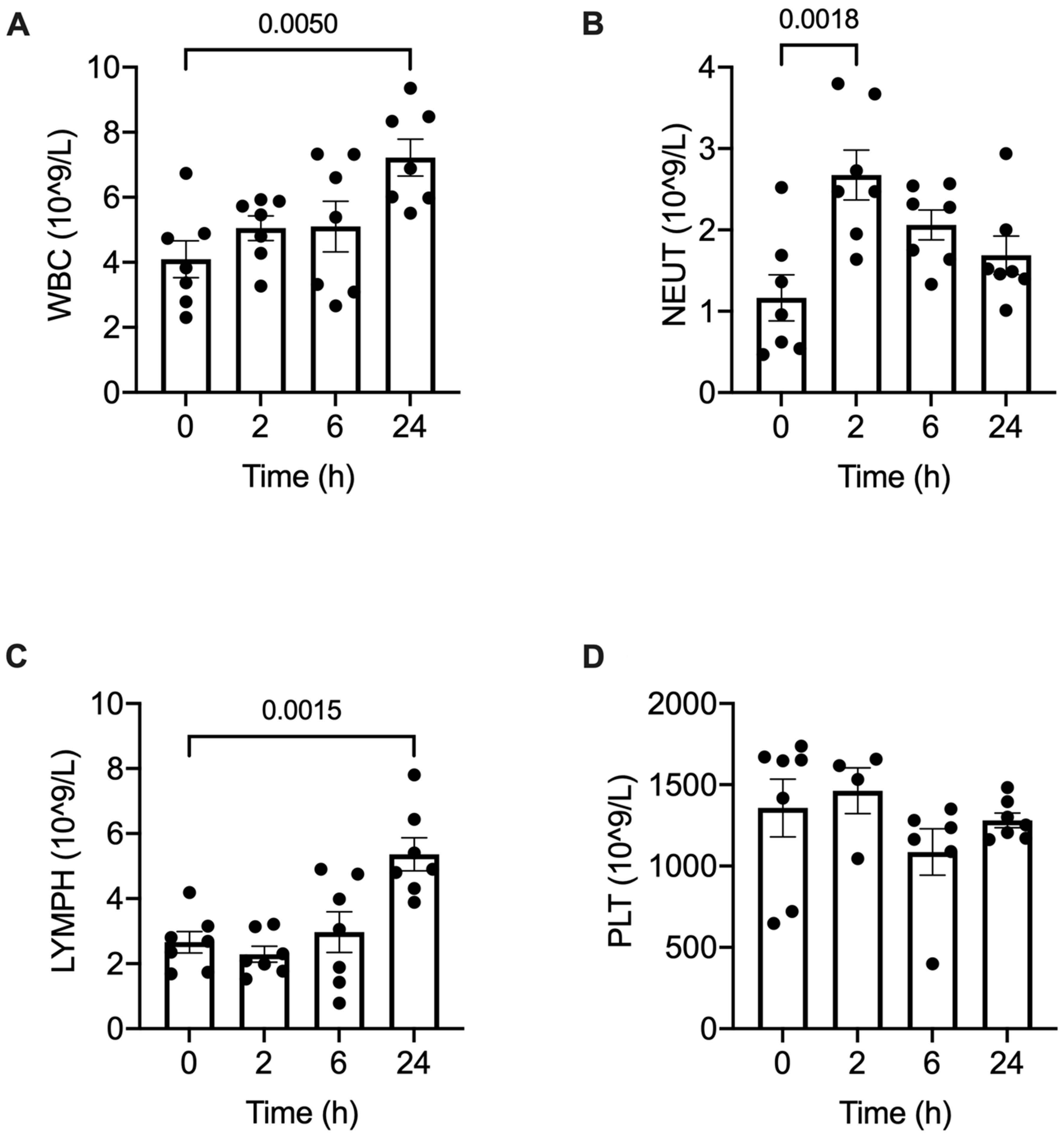

Supplemental Figure 9. Complete blood count after neutrophil transfusion.
Complete blood count was performed with blood harvested from the submandibular vein after 2, 6, and 24 hours of neutrophil infusion. (**A**) Total white cells (n=7). (**B**) Neutrophils (n=7). (**C**) Lymphocytes (n=7). (**D**) Platelets (n=3-7). P values from One-way ANOVA with Tukey correction.

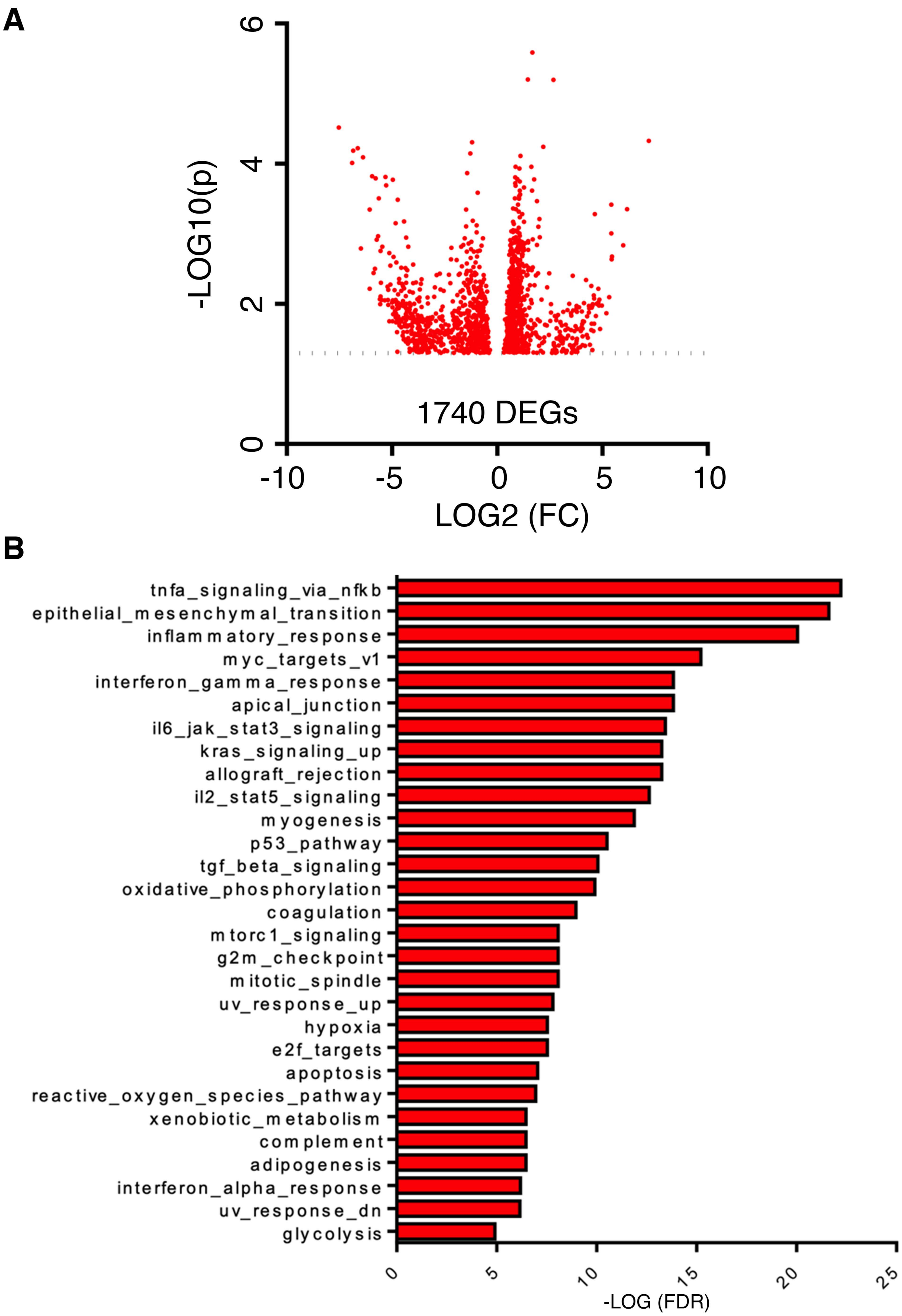

Supplemental Figure 10. RNA-seq study with cardiac neutrophils.
(**A**) Volcano plot showing 1740 differentially expressed genes (DEGs) between Cre and K2KO groups. The dashed line indicates p=0.05. (**B**) GSEA Hallmark pathway enrichment analysis of DEGs. Selected pathways are listed in Figure 9A. Cells sorted from four mice were included as biological replicates for each group (n=4). AngII infusion: 1 week.

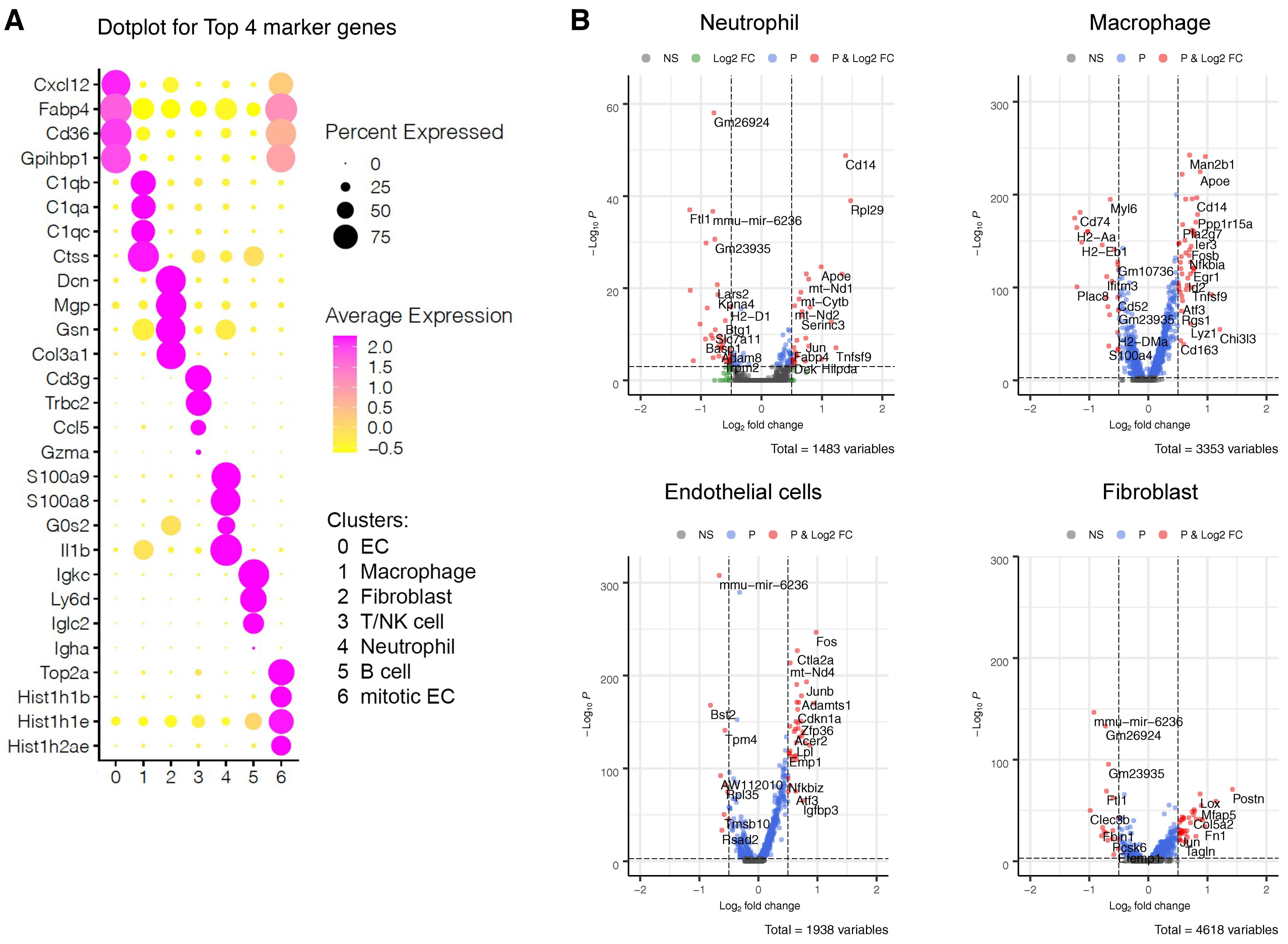

Supplemental Figure 11. Single-cell RNA-seq study with cardiac cells.
(**A**) Dotplot for Top 4 marker genes in each cluster. (**B**) Volcano plots showed DEGs in 4 major cell types identified from scRNA-seq. GO analysis was performed using these DEGs in Figure 11. Cells from three mice were pooled before flow sorting in each group (n=3). AngII infusion: 1 week.

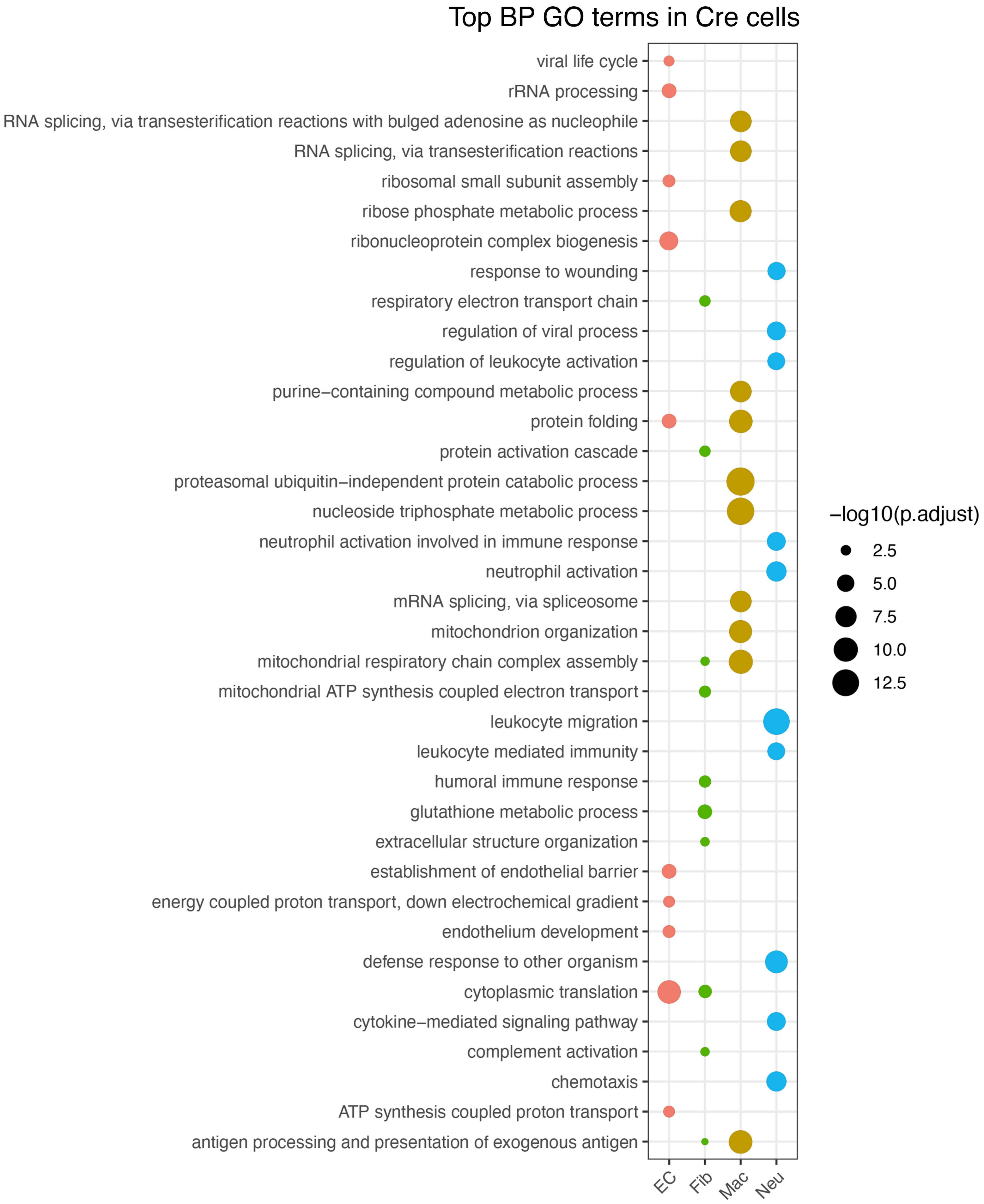

Supplemental Figure 12. Neutrophils orchestrate myocardial inflammation and adaptation to AngII stress. Gene ontology (GO) analyses with Cre DEGs from 4 major cell types: neutrophils, macrophages, endothelial cells, and fibroblasts, showing Top 10 Biological Process (BP) GO terms according to adjusted P values (p.adjust).

Supplementary Table 1. Patients' information.

Characteristic	Control (±SEM)	Heart Failure Patients (±SEM)
Number	8	8
Age (y)	75 ± 9.4	79 ± 8.8
Male sex (%)	50	75
LVEF (%)	61.5 ± 3.4	38.5 ± 8.05 ***
Pro-BNP(pg/mL)	199 ± 142	12317 ± 8823 *
Hypertrophy (Y/N)	N	Y

Characteristic	Control (±SEM)	Heart Failure Patients (±SEM)
Number	8	7
Age (y)	71± 9.4	70.3 ± 12.4
Male sex (%)	12.5	85.7
LVEF (%)	62.4 ± 4.69	41.1 ± 5.24 ***
Pro-BNP(pg/mL)	258.95 ± 329.43	3737 ± 3483 **
Hypertrophy (Y/N)	N	Y

*P < 0.05; **P < 0.01; ***P < 0.001 by 2-tailed unpaired Student t test.

Supplementary Table 2. Echocardiogram parameters.

Figure 2B

		sham		AngII	
Measurement	Units	Lyz2-Cre	Lyz2-K2KO	Lyz2-Cre	Lyz2-K2KO
IVS;d	mm	0.80 ± 0.09	0.77 ± 0.12	0.89 ± 0.04	0.87 ± 0.02
LVID;d	mm	3.36 ± 0.36	3.89 ± 0.26	3.92 ± 0.25	4.41 ± 0.45
LVPW;d	mm	0.80 ± 0.08	0.92 ± 0.09	1.18 ± 0.13	1.14 ± 0.10
IVS;s	mm	1.17 ± 0.11	1.20 ± 0.22	1.31 ± 0.09	1.02 ± 0.10***
LVID;s	mm	2.01 ± 0.19	2.31 ± 0.13	2.41 ± 0.10	3.34 ± 0.75*
LVPW;s	mm	1.22 ± 0.08	1.30 ± 0.12	1.39 ± 0.13	1.38 ± 0.11
LV Vol;d	ul	46.64 ± 12.67	66.08 ± 10.46	67.03 ± 9.78	89.17 ± 22.43
LV Vol;s	ul	13.14 ± 3.46	18.42 ± 2.54	20.53 ± 2.14	48.57 ± 30.08
%EF	%	71.62 ± 3.94	71.81 ± 2.37	68.96 ± 3.93	48.33 ± 16.84*
% FS	%	39.88 ± 2.65	40.63 ± 2.02	38.32 ± 3.18	24.78 ± 9.23*
LV Mass	mg	88.73 ± 16.71	123.33 ± 33.14	165.19 ± 30.06	189.22 ± 38.31
LV Mass Corrected	mg	70.98 ± 13.37	98.66 ± 26.51	132.15 ± 24.05	151.38 ± 30.65

*p<0.05, **p<0.01, ***p<0.001. AngII-Lyz2-Cre *vs.* AngII-Lyz2-K2KO
Data shown as Mean ± SD, N: as indicated in related figure panel.

Figure 3A

		K2KO	K2KO + AngII	
Measurement	Units	Sham	IgG	Ly6G
IVS;d	mm	0.79 ± 0.10	0.94 ± 0.05	0.88 ± 0.049
LVID;d	mm	3.54 ± 0.42	4.43 ± 0.32	3.86 ± 0.227*
LVPW;d	mm	0.73 ± 0.12	1.03 ± 0.25	0.98 ± 0.12
IVS;s	mm	1.06 ± 0.22	1.27 ± 0.25	1.22 ± 0.03
LVID;s	mm	2.18 ± 0.21	3.17 ± 0.18	2.34 ± 0.22***
LVPW;s	mm	1.1325 ± 0.12	1.15 ± 0.13	1.44 ± 0.16*
LV Vol;d	ul	53.16 ± 14.67	89.40 ± 15.02	64.29 ± 9.39*
LV Vol;s	ul	16.06 ± 3.73	40.08 ± 5.73	19.15 ± 4.41***
%EF	%	69.47 ± 2.69	54.80 ± 4.95	70.18 ± 5.89**
% FS	%	38.25 ± 2.36	28.35 ± 3.31	39.26 ± 4.67**
LV Mass	mg	89.98 ± 17.35	186.87 ± 49.81	136.49 ± 16.73
LV Mass Corrected	mg	71.98 ± 13.38	149.50 ± 39.85	109.19 ± 13.38

*p<0.05, **p<0.01, ***p<0.001 by 2-tailed unpaired Student t test. AngII-IgG *vs.* AngII-Ly6G
Data shown as Mean ± SD, N: as indicated in related figure panel.

**Figure 4B**		K2KO + AngII	
Measurement	Units	Vehicle	DNase I
IVS;d	mm	0.94 ± 0.14	0.89 ± 0.15
LVID;d	mm	3.92 ± 0.36	3.80 ± 0.23
LVPW;d	mm	1.08 ± 0.36	1.02 ± 0.10
IVS;s	mm	1.20 ± 0.17	1.24 ± 0.23
LVID;s	mm	2.63 ± 0.36	2.28 ± 0.23*
LVPW;s	mm	1.38 ± 0.39	1.58 ± 0.15
LV Vol;d	ul	67.64 ± 14.62	62.19 ± 9.23
LV Vol;s	ul	26.03 ± 8.85	17.89 ± 4.53*
%EF	%	61.79 ± 9.97	71.38 ± 4.89*
% FS	%	33.09 ± 6.58	40.16 ± 4.05*
LV Mass	mg	159.57 ± 44.32	138.78 ± 19.98
LV Mass Corrected	mg	127.65 ± 35.46	111.03 ± 15.98

*p<0.05, **p<0.01, ***p<0.001 by 2-tailed unpaired Student t test. AngII-Veh *vs.* AngII-Dnase I
Data shown as Mean ± SD, N: as indicated in related figure panel.

**Figure 4E**		K2KO + AngII	
Measurement	Units	Veh	GSK-484
IVS;d	mm	0.89 ± 0.12	0.79 ± 0.10
LVID;d	mm	3.92 ± 0.25	3.61 ± 0.24*
LVPW;d	mm	1.06 ± 0.16	1.03 ± 0.14
IVS;s	mm	1.05 ± 0.16	1.13 ± 0.11
LVID;s	mm	2.78 ± 0.30	2.13 ± 0.18***
LVPW;s	mm	1.24 ± 0.16	1.40 ± 0.14*
LV Vol;d	ul	67.38 ± 10.94	55.35 ± 8.86*
LV Vol;s	ul	30.21 ± 8.34	14.81 ± 3.31***
%EF	%	55.51 ± 8.14	73.21 ± 4.37***
% FS	%	28.78 ± 5.36	41.60 ± 3.67***
LV Mass	mg	151.06 ± 26.63	124.33 ± 22.12
LV Mass Corrected	mg	120.85 ± 21.31	99.46 ± 17.70

*p<0.05, **p<0.01, ***p<0.001 by 2-tailed unpaired Student t test. AngII-Veh *vs.* AngII-GSK-484
Data shown as Mean ± SD, N: as indicated in related figure panel.

**Figure 7A**		K2KO	K2KO + AngII	
Measurement	Units	Sham	Veh	Heparin
IVS;d	mm	0.66 ± 0.07	0.82 ± 0.11	0.77 ± 0.08
LVID;d	mm	3.97 ± 0.13	3.76 ± 0.15	3.79 ± 0.17
LVPW;d	mm	0.89 ± 0.21	1.14 ± 0.25	1.04 ± 0.23
IVS;s	mm	1.02 ± 0.16	0.81 ± 0.14	1.07 ± 0.09**
LVID;s	mm	2.44 ± 0.15	2.60 ± 0.17	2.05 ± 0.13***
LVPW;s	mm	1.34 ± 0.08	1.42 ± 0.31	1.40 ± 0.14
LV Vol;d	ul	68.63 ± 5.43	60.51 ± 5.56	61.90 ± 6.62
LV Vol;s	ul	21.26 ± 3.49	24.83 ± 4.09	13.62 ± 2.11***
%EF	%	69.25 ± 2.81	59.02 ± 4.81	77.77 ± 4.74***
% FS	%	38.52 ± 2.13	30.77 ± 3.30	45.90 ± 4.40***
LV Mass	mg	113.16 ± 25.18	141.61 ± 26.31	128.17 ± 22.63
LV Mass Corrected	mg	90.53 ± 20.15	113.29 ± 21.05	102.53 ± 18.11

*p<0.05, **p<0.01, ***p<0.001 by 2-tailed unpaired Student t test. AngII-Veh *vs.* AngII-Heparin
Data shown as Mean ± SD, N: as indicated in related figure panel.

**Figure 8A**		WT	Neutrophil transfusion	
Measurement	Units	AngII	AngII	DNase I + AngII
IVS;d	mm	0.80 ± 0.02	0.80 ± 0.06	0.76 ± 0.10
LVID;d	mm	3.52 ± 0.09	3.76 ± 0.19	3.66 ± 0.10
LVPW;d	mm	0.91 ± 0.10	0.94 ± 0.09	0.94 ± 0.08
IVS;s	mm	1.07 ± 0.06	0.94 ± 0.07	1.03 ± 0.11
LVID;s	mm	2.12 ± 0.19	2.67 ± 0.29	2.34 ± 0.13**
LVPW;s	mm	1.37 ± 0.11	1.22 ± 0.09	1.26 ± 0.11
LV Vol;d	ul	51.73 ± 3.32	60.64 ± 7.27	56.54 ± 3.77
LV Vol;s	ul	15.16 ± 3.26	26.67 ± 6.97	19.02 ± 2.60**
%EF	%	70.92 ± 6.18	56.53 ± 7.34	66.32 ± 4.40**
% FS	%	39.80 ± 5.17	29.23 ± 4.70	36.00 ± 3.36**
LV Mass	mg	105.94 ± 9.52	119.19 ± 15.19	110.59 ± 14.28
LV Mass Corrected	mg	84.75 ± 7.61	95.35 ± 12.16	88.47 ± 11.42

*p<0.05, **p<0.01, ***p<0.001 by 2-tailed unpaired Student t test. Neutrophil transfusion: AngII-Veh *vs.* AngII-Dnase I
Data shown as Mean ± SD, N: as indicated in related figure panel.

**Figure 9D**		AngII	
Measurement	Units	Lyz2-Cre	DKO
IVS;d	mm	0.85 ± 0.04	0.97 ± 0.21
LVID;d	mm	3.72 ± 0.30	3.48 ± 0.25
LVPW;d	mm	0.94 ± 0.13	1.06 ± 0.11
IVS;s	mm	1.27 ± 0.18	1.40 ± 0.20
LVID;s	mm	2.10 ± 0.19	2.02 ± 0.23
LVPW;s	mm	1.44 ± 0.21	1.37 ± 0.22
LV Vol;d	ul	59.51 ± 11.74	50.39 ± 8.62
LV Vol;s	ul	14.60 ± 3.44	13.28 ± 3.96
%EF	%	75.46 ± 3.22	73.03 ± 8.30
% FS	%	43.57 ± 3.02	41.68 ± 7.89
LV Mass	mg	123.55 ± 30.05	130.85 ± 17.03
LV Mass Corrected	mg	98.84 ± 24.03	104.675 ± 13.62

*p<0.05, **p<0.01, ***p<0.001 by 2-tailed unpaired Student t test. AngII-Lyz2-Cre *vs* . AngII-DKO
Data shown as Mean ± SD, N: as indicated in related figure panel.

**Figure S3**		Sham		AngII	
Measurement	Units	Cx3cr1-Cre	Cx3cr1-K2KO	Cx3cr1-Cre	Cx3cr1-K2KO
IVS;d	mm	0.82 ± 0.04	0.80 ± 0.09	0.96 ± 0.07	0.88 ± 0.06
LVID;d	mm	3.26 ± 0.20	3.33 ± 0.19	3.60 ± 0.06	3.60 ± 0.20
LVPW;d	mm	0.82 ± 0.06	0.75 ± 0.10	1.04 ± 0.22	0.92 ± 0.16
IVS;s	mm	1.19 ± 0.06	1.01 ± 0.12	1.25 ± 0.16	1.25 ± 0.27
LVID;s	mm	1.87 ± 0.18	1.96 ± 0.26	2.06 ± 0.12	2.06 ± 0.09
LVPW;s	mm	1.29 ± 0.05	1.17 ± 0.11	1.46 ± 0.26	1.33 ± 0.01
LV Vol;d	ul	42.92 ± 6.37	45.47 ± 6.73	54.50 ± 2.29	54.54 ± 7.12
LV Vol;s	ul	10.88 ± 2.55	12.39 ± 4.24	13.86 ± 1.96	13.70 ± 1.56
%EF	%	74.75 ± 4.07	73.16 ± 6.46	74.43 ± 4.59	74.64 ± 4.00
% FS	%	42.55 ± 3.60	41.34 ± 5.45	42.61 ± 4.17	42.73 ± 3.69
LV Mass	mg	87.03 ± 9.85	83.16 ± 12.57	135.35 ± 14.54	116.80 ± 15.22
LV Mass Corrected	mg	69.62 ± 7.88	66.53 ± 10.06	108.28 ± 11.64	93.44 ± 12.18

*p<0.05, **p<0.01, ***p<0.001 by 2-tailed unpaired Student t test. AngII-Cx3cr1-Cre *vs* . AngII-Cx3cr1-K2KO
Data shown as Mean ± SD, N: as indicated in related figure panel.

Figure S7

Measurement	Units	WT + AngII 2.0	
		IgG	Ly6G
IVS;d	mm	0.94 ± 0.05	0.88 ± 0.05
LVID;d	mm	4.43 ± 0.32	3.86 ± 0.23*
LVPW;d	mm	1.03 ± 0.25	0.99 ± 0.12
IVS;s	mm	1.27 ± 0.25	1.23 ± 0.03
LVID;s	mm	3.17 ± 0.18	2.34 ± 0.22***
LVPW;s	mm	1.15 ± 0.13	1.44 ± 0.16*
LV Vol;d	ul	89.40 ± 15.02	64.29 ± 9.39*
LV Vol;s	ul	40.08 ± 5.73	19.15 ± 4.41***
%EF	%	54.80 ± 4.95	70.18 ± 5.89**
% FS	%	28.34 ± 3.32	39.27 ± 4.67**
LV Mass	mg	186.88 ± 49.81	136.50 ± 16.73
LV Mass Corrected	mg	149.50 ± 39.85	109.20 ± 13.38

*p<0.05, **p<0.01, ***p<0.001 by 2-tailed unpaired Student t test. AngII-IgG *vs* . AngII-Ly6G
Data shown as Mean ± SD, N: as indicated in related figure panel.
